# *miR-370-3p* Inhibited the Proliferation of Sheep Dermal Papilla Cells by Inhibiting the Expression of *SMAD4*

**DOI:** 10.3390/cells14100714

**Published:** 2025-05-14

**Authors:** Jiaqi Fu, Dan Wang, Wenqing Liu, Yu Qi, Caihong Zhang, Huansong Li, Jinshun Cai, Shuang Ji, Lichun Zhang, Fuliang Sun

**Affiliations:** 1 College of Agriculture, Yanbian University, Yanji 133002, China; 2022010659@ybu.edu.cn (J.F.); wangdan.1118@foxmail.com (D.W.); 2023010683@ybu.edu.cn (W.L.); qiyu3630@163.com (Y.Q.); zch2023010692@126.com (C.Z.); 18664282041@163.com (H.L.); jinshuncai@163.com (J.C.); jishuang@ybu.edu.cn (S.J.); 2 Animal Disease Prevention and Control Center of Panshi City, Panshi 132300, China; 3 Animal Biotechnology Institute, Jilin Academy of Agricultural Sciences, Gongzhuling 136100, China

**Keywords:** *miR-370-3p*, *SMAD4*, dermal papilla cells, cell phenotype

## Abstract

The proliferation and maturation of hair follicles in follicular papilla cells are predominantly governed by miRNAs, which significantly influence the cell cycle, apoptosis, and proliferation. *miR-370-3p* has been associated with several biological processes and targets *SMAD4*, a crucial component in hair follicle development. Tissue expression profiling revealed significant differences in *miR-370-3p* levels between skin tissues of the two sheep breeds in January and October, as well as between tissues of the Xinji fine-wool sheep and Small-tail Han sheep. *SMAD4* exhibited significant differences in tissue-specific expression in the heart, spleen, skin, lungs, and muscles from Xinji fine-wool sheep and Small-tail Han sheep. Bioinformatics analysis and dual-luciferase reporter assays validated the regulatory interaction between *miR-370-3p* and *SMAD4*. CCK-8 experiments demonstrated that *miR-370-3p*’s targeting of *SMAD4* suppressed cell growth. Cell cycle analysis demonstrated that *miR-370-3p*’s targeting of *SMAD4* influenced the cell cycle. Annexin V-FITC/PI dual labeling demonstrated that *miR-370-3p*’s targeting of *SMAD4* promoted cell apoptosis. RT-qPCR data demonstrated that *miR-370-3p*’s targeting of *SMAD4* elevated the expression of *JUN*, *c-MYC*, and *TCF7L2* while suppressing β-catenin expression. Western blot (WB) analysis demonstrated that *miR-370-3p* targeting of *SMAD4* significantly promoted c-MYC expression while inhibiting CCND1, CCND2, and β-catenin expression. *miR-370-3p* and *SMAD4* exhibit spatiotemporal expression differences in sheep skin tissues, with widespread expression across various tissues. Furthermore, it confirmed that *miR-370-3p* targets *SMAD4* to inhibit follicular papilla cell proliferation, promote apoptosis, and influence the cell cycle.

## 1. Introduction

Hair follicles are specialized micro-organs with complex structures and distinct functions, extending from the follicular bulge to the basal region and consisting of integral components such as the hair germ and dermal papilla (DP) [1]. The hair follicle passes through several cyclic phases, including the anagen, catagen, and telogen [2], each characterized by distinct tissue and physiological features governed by extensive gene activation and silencing. The shift from telogen to anagen is characterized by the initiation of new hair shaft formations and the activation of many signaling pathways that regulate the expression of genes related to hair-specific keratin production, inner root sheath development, and pigmentation [3].

MicroRNAs (miRNAs) are small, non-coding RNA molecules, generally around 24 nucleotides long, that are generated endogenously by cells. These molecules attach to complementary sequences in the 3′ untranslated region (3′UTR) of target mRNAs to inhibit translational expression, elucidating its regulatory role [4]. Research has shown that microRNAs (miRNAs) are essential regulators of hair follicle growth and development. *miR-205* exhibits significant differences between growth and regression phases in mice, influencing hair follicle cycle transitions [5]. Cao et al. posited that *miR-100* derived from exosome-mimetic nanovesicles (ReN-NVs) produced by neural progenitor cells enhances nuclear *β*-*catenin* expression, inhibits several Wnt negative regulators, and elevates *C-myc* and *Cyclin D1* (*CCND1*) levels, thereby facilitating the acceleration of hair follicle growth. Additionally, *miR-99/100* and let-7a/c have been identified as pivotal regulators for the regeneration of cardiomyocytes [6]. Moreover, it has been established that *miR-218* regulates the Wnt signaling pathway by inhibiting the expression of the *SFRP2* gene, consequently affecting apoptosis and resulting in significant variations in rabbit hair length. This finding provides a foundation for future investigations on *miR-218*′s function in hair follicle growth [7].

In our prior research, we sequenced differentially expressed miRNAs from the skin tissues of Xinji fine-wool sheep and Small-tail Han sheep, each exhibiting unique wool characteristics, and revealed the differential expression of *miR-370-3p*. Bioinformatic investigations indicated that SMAD family member 4 (*SMAD4*) may be a target gene of *miR-370-3p*. Moreover, a mechanism underlying the growth/rest switch in hair follicles has been proposed, which involves endothelial glycogen-dependent cross-signaling between the Wnt/β-catenin and BMP/Smad pathways [8]. Studies have already shown that *SMAD4* and *SMAD7* are essential regulators of the formation and development of hair follicles. The bone morphogenetic protein (BMP) signaling pathway primarily mediates the effect of *SMAD4* on follicular development, whereas *SMAD7* disrupts TGF-β/activin/BMP signaling and promotes ubiquitin-mediated degradation of β-catenin, hence inhibiting the Wnt/β-catenin signaling cascade. These activities substantially influence follicular growth and differentiation, and their study demonstrated that *SMAD2* and *SMAD4* cooperatively regulate epidermal cell homeostasis via the TGF-β pathway [9]. Owens et al. suggested that the loss of the *SMAD4* gene contributes to partial degeneration of hair follicles in the skin [10], while Yang et al. highlighted the critical function of *SMAD4* in maintaining the shape of hair follicle stem cells [11]. Yang et al. [11] also highlighted the vital role of *SMAD4* in preserving the structural integrity of hair follicle stem cells, whereas Owens et al. [10] proposed that the absence of the *SMAD4* gene leads to the deterioration of cutaneous hair follicles.

This study aimed to confirm the relationship between *miR-370-3p* and its target gene *SMAD4* and to explore the specific mechanism by which they regulate hair follicle growth, development, and cycling, providing a theoretical basis for the miRNA regulation of dermal papilla cell (DPC) growth and development.

## 2. Materials and Methods

### 2.1. Ethical Statement

Three healthy Xinji fine-wool sheep and three healthy Small-tail Han sheep of the same age were selected from the Animal Husbandry Branch of Jilin Academy of Agricultural Sciences. Professional experimenters conducted regular checks on the health status of the experimental animals. To minimize environmental influences, both groups of sheep were kept in the same environment and fed with the same feed. Skin tissue samples were harvested during the anagen phase (October) and the telogen phase (January) of the hair follicle cycle for examination. The livestock collection was carried out following the regulations authorized by the Animal Welfare and Ethics Committee of Jilin Academy of Agricultural Sciences (JNK20210901001, 11 September 2021). Moreover, the international animal welfare guidelines were strictly followed, and euthanasia was performed on the experimental sheep. The drug used for euthanasia was sodium pentobarbital at a dosage of 120 mg/kg. All of the euthanasia procedures and sample collection were conducted by professionally trained and experienced experimental personnel to ensure the accuracy of the operation process and minimize pain for the animals.

### 2.2. Sample Collection

One-year-old female Small-tail Han and Xinji fine-wool sheep that were not in estrous were used in this study. They were chosen from the Jilin Academy of Agricultural Sciences’ Animal Husbandry Institute. After shearing the wool around the scapular region, the area was disinfected with 75% alcohol. Local anesthesia was administered by subcutaneous injection of lidocaine hydrochloride (Jianmin Pharmaceuticals, Wuhan, China). Under the guidance of professional experimenters, three 1-cm^2^ skin tissue samples were collected from each sheep. These three skin tissue samples were used for different experiments. The first skin tissue samples were placed in PBS containing 1% penicillin/streptomycin (Bioss, Beijing, China) and stored at 4 °C for DPC isolation. The second skin tissue samples were preserved in 4% paraformaldehyde (Biosharp, Hefei, China) and subsequently stored at room temperature for immunohistochemical analysis. In the third set of samples, the skin, heart, liver, spleen, lungs, kidneys, and muscle tissues were promptly snap-frozen in liquid nitrogen and stored at −80 °C for total RNA and miRNA isolation.

### 2.3. DPC Isolation and Culture

The skin tissues were subjected to washing with 75% ethanol, followed by three consecutive rinses with PBS containing 1% penicillin/streptomycin. This procedure was performed a total of three times. After washing, the hair shafts and subcutaneous fat layers were removed. DPCs from Small-tail Han sheep were isolated, cultured, and characterized in accordance with the protocols previously developed in our laboratory [12]. The cells were kept in a CO_2_ incubator (Thermo Fisher Scientific, Waltham, MA, USA) at 37 °C with 5% CO_2_ in DMEM/F12 (BI, Shanghai, China) supplemented with 10% fetal bovine serum (Cell-Box, Hong Kong, China) and 1% penicillin/streptomycin solution.

### 2.4. RNA Extraction and Quantitative Real-Time PCR (qRT-PCR)

The RNAeasyTM Animal RNA Extraction Kit (centrifugal column method) (Beyotime, Shanghai, China) was utilized for total RNA extraction. The concentration and purity of the RNA samples were evaluated using a spectrophotometer (Thermo Fisher Scientific, Waltham, MA, USA); and all samples tested had OD_260/280_ values of 1.8–2.0. Following this, genomic DNA was eliminated, and first-strand cDNA was produced with the HiFiScript Quick gDNA Removal cDNA Synthesis Kit (Cwbio, Taizhou, China), and the resultant cDNA was preserved at −20 °C. Gene expression measurement was conducted with the GoTaq^®^ qPCR Master Mix (Promega, Beijing, China). The 20 μL reaction mixture comprised 10 μL of 2 × GoTaq^®^ qPCR Master Mix, 0.4 μL of each forward and reverse primer, 2 μL of cDNA, and 7.2 μL of Nuclease-Free Water, Waltham, MA, USA. The PCR amplification was performed using the following parameters: an initial denaturation at 95 °C for 10 min, followed by 45 cycles of denaturation at 95 °C for 15 s, and annealing/extension at 60 °C for 1 min. β-actin was employed as the internal reference gene for normalization in the qPCR analysis.

Total miRNA was isolated using the miRcute miRNA Isolation Kit (TIANGEN, Beijing, China), according to the manufacturer’s instructions to ensure the efficient recovery of high-quality miRNA from tissue samples. First-strand cDNA was synthesized using the miRcute Enhanced miRNA cDNA First Strand Synthesis Kit (TIANGEN, Beijing, China), according to the manufacturer’s guidelines. The generated cDNA was subsequently preserved at −20 °C for future examination. The expression of miRNA was measured using the miRcute Enhanced miRNA Fluorescence Quantification Kit (TIANGEN, Beijing, China), adhering to the manufacturer’s guidelines, to precisely assess miRNA levels in the samples. The 20 μL reaction mixture consisted of 10 μL of 2 × PreMix (SYBR and ROX), 0.4 μL of forward primer (200 nM), 0.4 μL of reverse primer (200 nM), 1 μL of cDNA, and 8.2 μL of nuclease-free water. The PCR amplification method comprises an initial denaturation step at 95 °C for 15 min, followed by 5 cycles of denaturation at 94 °C for 20 s, annealing at 65 °C for 30 s, and elongation at 72 °C for 34 s. This was followed by 45 cycles of denaturation at 94 °C for 20 s and annealing at 60 °C for 34 s. U6 small nuclear RNA functioned as the internal control, utilizing the U6 forward primer sourced from Sangon Biotech (Shanghai, China) to normalize the miRNA expression data and reduce any variations in RNA input.

The expression levels of the target genes or miRNAs were quantified using the 2^−ΔΔCt^ method. All primers employed for qRT-PCR are listed in Table 1.

### 2.5. 3′-UTR Luciferase Reporter Assay

The target genes of *miR-370-3p* were identified utilizing four internet platforms: miRDB, miRWalk, TargetScan, and starBase. We selected the intersection of the prediction results from these four platforms, and among the genes in the intersection, *SMAD4* was chosen for further investigation. The protein–protein interactions of *SMAD4* were examined utilizing the web program STRING, and an miRNA–mRNA–mRNA interaction network was developed with Cytoscape 3.9.1.

The interaction sites between *miR-370-3p* and *SMAD4* were predicted using RNAhybrid. According to the expected binding sites, plasmids were designed and constructed: *miR-370-3p*-mimics, mimics negative control (NC), pmirglo-*miR-370-3p*-*SMAD4*-WT, and pmirglo-*miR-370-3p*-*SMAD4*-Mut. The constructs were co-transfected into HEK-293T cells using Lipofectamine 3000 (Invitrogen, Carlsbad, CA, USA). Samples were collected 48 h post-transfection. The assays employed a luciferase reporter gene kit (Promega, Beijing, China). The mutation sites are shown in Table 2.

### 2.6. Cell Transfection

The *miR-370-3p* mimic, mimic negative control (*miR-370-3p* mimic-NC), *miR-370-3p* inhibitor, and inhibitor negative control (*miR-370-3p* inhibitor-NC) (GenePharma, Shanghai, China) were transfected into DPCs utilizing Lipofectamine 2000 (Invitrogen, Carlsbad, CA, USA). DPCs were then uniformly seeded into a 6-well plate and allowed to proliferate until they reached 75% confluence with a complete growth medium. Transfection was subsequently executed utilizing Lipofectamine™ 2000 (all procedures were carried out under dim lighting). Subsequently, 3.75 μL of mimic or inhibitor was combined with 7.5 μL of Lipofectamine™ 2000 in 125 μL of Opti-MEM™ I reduced-serum medium and incubated at ambient temperature for 5 min. The Lipofectamine 2000 solution was thereafter mixed with the mimic or inhibitor combination and incubated at room temperature for 25 min. The incubated mixture was thereafter poured dropwise into each well of the 6-well plate, at a volume of 250 μL per well, and swirled gently. Subsequent to transfection, cells in the 6-well plates were cultured at 37 °C with 5% CO_2_ for 48 h before samples were collected for total RNA and total protein extraction.

siRNA-*SMAD4*, siRNA negative control (siRNA-NC), pOGP-T2A, and pOGP-T2A-*SMAD4* (pcDNA3.1, JST Scientific, Wuhan, China) were electroporated into DPCs using CUY21 EDIT II (BEX, Tokyo, Japan). Growth-arrested DPCs were seeded into culture bottles and cultured until reaching 90% confluence, with the complete medium being replaced by cell culture medium at 37 °C in a cell culture incubator with 5% CO_2_ for 24 h. Following two PBS rinses, the cells were digested with 0.25% trypsin, centrifuged for five minutes at 1500× *g*, and the supernatants removed. Cells were resuspended in 2 mL of Opti-MEM™ I reduced-serum medium, centrifuged at 1500× *g* for 5 min, and the supernatants were then discarded. Cells were then resuspended in 2 mL of Opti-MEM™ (Invitrogen, Carlsbad, CA, USA) I reduced-serum medium, stained with trypan blue, counted using a cell counter, and 1 × 10^6^ cells were removed and resuspended in 100 μL of Opti-MEM™ I reduced-serum medium. Subsequently, 1 μg of plasmid DNA was added, mixed thoroughly, and transferred into an electroporation cuvette. Electroporation parameters were set, and the cuvette was placed in the electroporator, and after verifying acceptable resistance, electroporation was performed. Post-electroporation, the cells were aspirated back into the culture bottle using a pipette and supplemented with cell culture medium. Fluorescence was detected using a fluorescence microscope after 24 h, and cells were collected 48 h post-electroporation for total RNA and total protein extraction. All sequences utilized are enumerated in Table 3.

### 2.7. Western Blot

Skin tissue samples were collected and immersed in liquid nitrogen prior to being pulverized in a mortar. Following the lysis of the powder using RIPA tissue/cell lysis buffer (Solarbio, Beijing, China) and subsequent storage at 4 °C, the supernatant was obtained using centrifugation at 2000 rpm for five minutes. The culture media were withdrawn after 48 h of cell transfection, and the cells were lysed using a pre-cooled RIPA lysis solution containing 1% PMSF. The supernatants were subsequently collected. The protein content was determined using the BCA Protein Assay Kit (Cwbio, Taizhou, China).

Proteins were fractionated by SDS-PAGE (10%) and subsequently transferred to PVDF membranes (Sigma Aldrich, Shanghai, China). Blocking was performed using 5% Blotting Grade (Beyotime, Shanghai, China). The chemiluminescent reaction was conducted using Super ECL Plus (UElandy, Suzhou, China), prepared according to a specified ratio. The primary antibodies utilized were SMAD4 (1:1000, Proteintech, Wuhan, China), β-actin (1:2000, Proteintech, Wuhan, China), CCND1 (1:10,000, Proteintech, Wuhan, China), CCND2 (1:1000, Proteintech, Wuhan, China), c-MYC (1:10,000, Proteintech, Wuhan, China), and β-catenin (1:10,000, Proteintech, Wuhan, China).

### 2.8. Cell Counting Kit-8 (CCK-8) Assay

DPCs were uniformly seeded in a 96-well plate at a density of 1 × 10^3^ cells per well. Cells underwent a 24 h incubation prior to transfection. Each well was administered 100 µL of fresh culture medium (DMEM/F12 + 10% FBS) supplemented with 10% CCK-8 (Beyotime, Shanghai, China) at 24, 48, 72, and 96 h post-transfection. The wells were subsequently incubated for two hours at 37 °C in darkness. A SpectraMax iD5 microplate reader (Molecular Devices, San Jose, CA, USA) was employed to measure absorbance at 450 nm to assess the reaction intensity and furnish quantitative data for further cellular response analysis.

### 2.9. Flow Cytometry Assay (FCM)

DPCs were inoculated in 6-well plates and grown until achieving 70–90% confluence prior to transfection. After 48 h of transfection, the progression of the cell cycle in DPCs was evaluated using a Cell Cycle and Apoptosis Detection Kit (Beyotime, Shanghai, China), following the manufacturer’s guidelines. Additionally, apoptosis in the DPCs was assessed using an Annexin V-FITC Apoptosis Detection Kit (Beyotime, Shanghai, China), following the provided protocol. Flow cytometry (Becton, Dickinson, and Company, Queensbury, NY, USA) was employed for comprehensive analysis of cell cycle distribution and apoptosis in DPCs following transfection, allowing for precise quantification and characterization of cellular responses.

### 2.10. Immunohistochemistry

Skin tissue samples from Xinji fine-wool sheep and Small-tail Han sheep were harvested during both the growth and resting phases and thereafter preserved in 4% paraformaldehyde (Biosharp, Hefei, China) for 48 h to preserve tissue morphology for subsequent analysis. After trimming to the appropriate size, the skin tissues were washed in embedding cassettes for 24 h. Following dehydration and paraffin embedding, sections were taken at ambient temperature. The slides were incubated in a 3% hydrogen peroxide solution (ANNJET, Dezhou, China) in the dark at ambient temperature for 25 min to suppress endogenous peroxidase activity. Thereafter, non-specific binding sites were blocked by incubating the slides with 3% BSA (Servicebio, Wuhan, China) at ambient temperature for 30 min. The *SMAD4* basic antibody (1:100, Proteintech, Wuhan, China) was diluted in PBS (Servicebio, Wuhan, China) and administered to the slides, followed by overnight incubation at 4 °C. Following PBS washing, the slides were incubated at room temperature for 50 min with an HRP-conjugated goat anti-mouse IgG secondary antibody (1:200, Servicebio, Wuhan, China). The reaction was visualized using DAB chromogenic reagent (Servicebio, Wuhan, China) under a microscope (E100, Nikon, Japan).

### 2.11. Statistical Analyses

All data are presented as mean ± standard deviation (SD). Statistical analyses were conducted using SPSS 26 software (IBM, Chicago, IL, USA), with significance evaluated by *t*-tests or one-way ANOVA, subsequently followed by LSD and Duncan’s post hoc tests. Graphical representations and data analysis were performed using GraphPad Prism 9, the latest version (GraphPad Software, San Diego, CA, USA). Differences were considered nonsignificant (ns) when *p* > 0.05, with * *p* < 0.05 indicating significance, ** *p* < 0.01 indicating high significance, *** *p* < 0.001 indicating very high significance, and **** *p* < 0.0001 indicating extreme significance.

## 3. Results

### 3.1. Differences in the Spatiotemporal Expression of miR-370-3p and SMAD4 mRNA in Sheep

**Figure 1:**
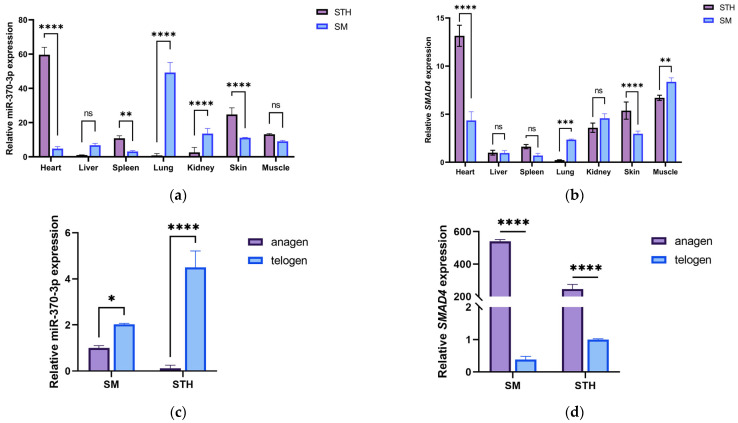
The expression patterns and spatiotemporal distribution of *miR-370-3p* and *SMAD4* in several tissues of Xinji fine-wool sheep and Small-tail Han sheep. (**a**) Distribution of *miR-370-3p* in various tissues of Xinji fine-wool sheep and Small-tail Han sheep. (**b**) Distribution of *SMAD4* mRNA in various tissues of Xinji fine-wool sheep and Small-tail Han sheep. (**c**) Expression levels of *miR-370-3p* in skin tissues across several sheep breeds at multiple time intervals. (**d**) Quantification of *SMAD4* expression levels in skin tissues across several sheep breeds at multiple time intervals. STH: Small-tail Han sheep; SM: Xinji fine-wool sheep; anagen: growing phase; telogen: resting phase. Data are shown as mean ± SD (n = 3). Comparisons within the same breed across several tissues: distinct superscript letters denote significant changes (*p* < 0.05); identical superscript letters signify no significant differences (*p* > 0.05). Comparative analysis of the same tissue across several breeds: * *p* < 0.05, ** *p* < 0.01, *** *p* < 0.001, **** *p* < 0.0001, ns: not significant.

We utilized qRT-PCR to evaluate the expression patterns of *miR-370-3p* and *SMAD4* mRNA in various tissues of both Xinji fine-wool sheep and Small-tail Han sheep, as illustrated in Figure 1. *miR-370-3p* exhibited extensive expression throughout seven tissues in both Small-tail Han sheep and Xinji fine-wool sheep, with peak expression levels recorded in the heart of Small-tail Han sheep and the lungs of Xinji fine-wool sheep. A comparative analysis demonstrated a significantly increased amount of *miR-370-3p* in the heart, spleen, and skin tissues of Small-tail Han sheep compared to Xinji fine-wool sheep, but expression was drastically reduced (Figure 1a). *SMAD4* mRNA exhibited extensive expression throughout the seven tissues in both sheep breeds, with peak expression observed in the heart of Small-tail Han sheep and diminished levels in the spleen, liver, and lungs. In contrast, the highest expression in Xinji fine-wool sheep was observed in muscle tissue, with lower expression in the liver and spleen. Comparative analysis demonstrated a notable upregulation of *SMAD4* mRNA expression in the heart, spleen, and skin tissues of Small-tail Han sheep relative to Xinji fine-wool sheep, while a noticeable downregulation of *SMAD4* expression was detected in the lungs and muscle tissues (Figure 1b).

Considering that *SMAD4* is a predicted target of *miR-370-3p*, we verified their expression levels in the skin tissues of Small-tail Han sheep and Xinji fine-wool sheep during the growth and telogen phases by RT-qPCR. The results showed that the expression level of *miR-370-3p* was low in the skin tissues of the two types of sheep during the growth phase, while the expression pattern of *SMAD4* was opposite to that of *miR-370-3p*. This indirectly indicates a targeting relationship between *miR-370-3p* and *SMAD4* (Figure 1c,d).

### 3.2. Localization of SMAD4 in Different Skin Tissues

**Figure 2:**
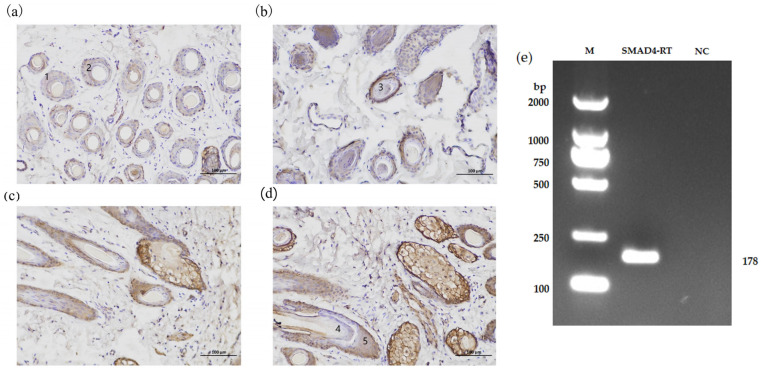
Localization of *SMAD4* in skin tissues and its expression in DPCs. (**a**) The cross-section of tissue from the skin from Xinji fine-wool sheep. (**b**) Cross-section of skin tissue from Small-tail Han sheep. (**c**) Longitudinal slice of dermal tissue from Xinji fine-wool sheep. (**d**) Longitudinal slice of dermal tissue from Small-tail Han sheep. 1—Connective tissue sheath; 2—Outer root sheath; 3—Hair cortex; 4—Hair bulb; 5—Dermal sheath. (**e**) Expression of *SMAD4* in DPCs. M D2000 DNA Marker; *SMAD4*-RT *SMAD4* gene PCR amplification product; NC negative control.

Immunohistochemical analysis showed the presence and distribution of *SMAD4* protein in the hair follicles of both Xinji fine-wool sheep and Small-tail Han sheep. Cross-sectional examination demonstrated expression in the connective tissue sheath, outer root sheath, and hair cortex (Figure 2a,b), whereas longitudinal sections exhibited expression in the hair bulb and dermal sheath (Figure 2c,d). Based on these findings, it is concluded that *SMAD4* protein is expressed in the DPCs. PCR using DPC cDNA as a template confirmed the expression of the *SMAD4* gene in DPCs (Figure 2e and Figure S1).

### 3.3. Binding of miR-370-3p to the 3′UTR of SMAD4

**Figure 3:**
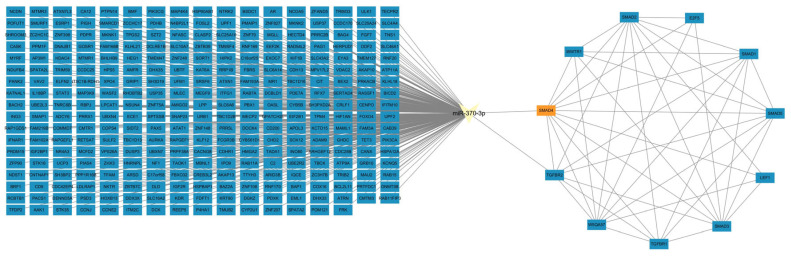
Network Interaction Analysis for *miR-370-3p* and *SMAD4* mRNA.

**Figure 4:**
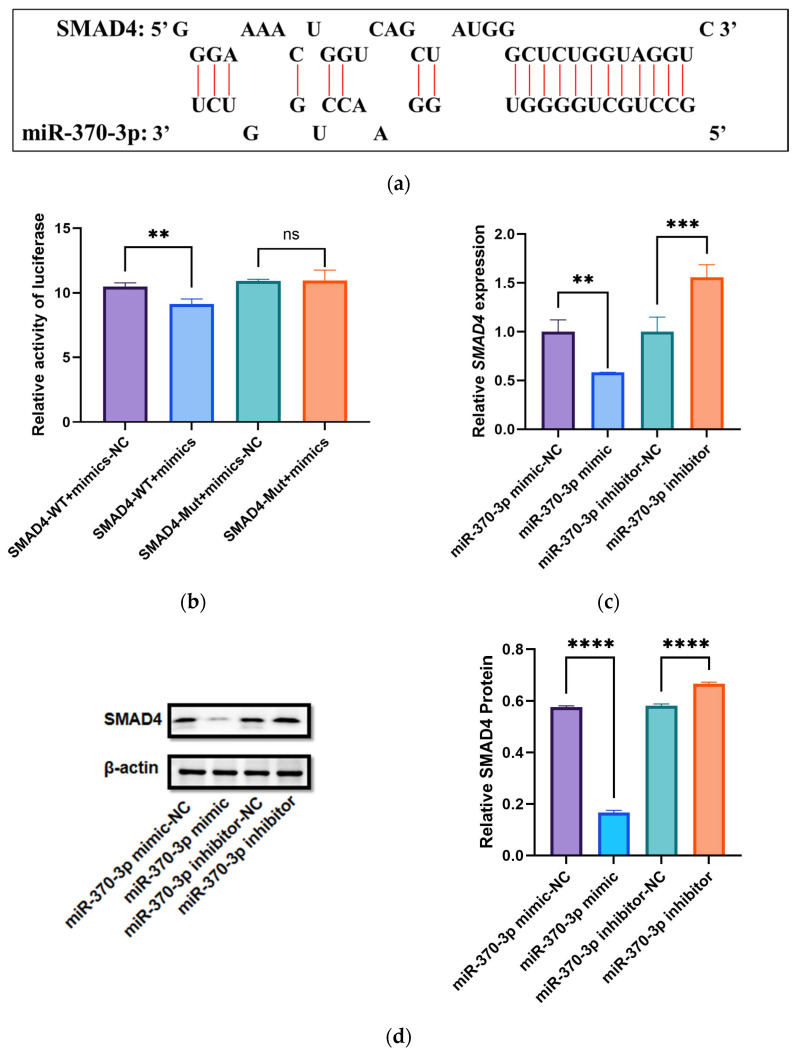
Validation of the correlation between *miR-370-3p* and *SMAD4*. (**a**) Illustration of the binding site for the target gene. (**b**) Relative luciferase activity following transfection with mimic NC combined with pmirglo-oar-*miR-370-3p*-*SMAD4*-WT, *miR-370-3p* mimics with pmirglo-oar-*miR-370-3p*-*SMAD4*-WT, mimic NC with pmirglo-oar-*miR-370-3p*-*SMAD4*-Mut, and *miR-370-3p* mimics with pmirglo-oar-*miR-370-3p*-*SMAD4*-Mut for a duration of 48 h. (**c**) The impact of *miR-370-3p* mimetics and antagonists on the mRNA expression levels of *SMAD4*. (**d**) Impact of *miR-370-3p* on the expression levels of *SMAD4* protein; Grayscale evaluation of *SMAD4* protein expression. Data are shown as mean ± standard deviation (n = 3). Statistical significance is shown by ** *p* < 0.01, *** *p* < 0.001, **** *p* < 0.0001, while “ns” signifies no significant difference.

The results of target gene prediction indicated that *miR-370-3p* may have a targeting relationship with *SMAD4*. The results of protein interaction prediction showed that *SMAD4* interacts with *SMAD2*, *SMAD1*, *TGFBR1*, and other proteins (Figure 3). To ascertain if *miR-370-3p* targets the *SMAD4* gene, RNAhybrid was employed to predict binding sites, demonstrating complementary interaction between the mature sequencing of *miR-370-3p* and the 3′UTR of *SMAD4* (Figure 4a). Based on these predicted binding sites, wild-type pmirGLO vectors containing the 3′UTR region of *SMAD4* (*SMAD4*-WT) and a mutant vector (*SMAD4*-Mut) were generated and subsequently transfected into HEK-293T cells. After 48 h, relative luciferase activity (RLU) was markedly diminished in the WT group (pmirglo-oar-*miR-370-3p*-*SMAD4*-WT), contrasted with the control group, but no notable difference was seen in the MUT group (mimics NC + pmirglo-oar-*miR-370-3p*-*SMAD4*-Mut) in relation to the control (Figure 4b). The results demonstrate that *miR-370-3p* directly interacts with *SMAD4* by adhering to its 3′ untranslated region (UTR). The findings demonstrate that *SMAD4* is an exact target gene of *miR-370-3p*.

DPCs were incubated with *miR-370-3p* mimics, mimic NC, *miR-370-3p* inhibitor, and inhibitor NC for 48 h, and subsequently subjected to RT-qPCR and Western blot analyses to evaluate the regulatory effect of *miR-370-3p* on the mRNA and protein expression levels of *SMAD4*.RT-qPCR research revealed a substantial downregulation of *SMAD4* expression following the overexpression of *miR-370-3p*, whereas the silencing of *miR-370-3p* led to a pronounced upregulation of *SMAD4* expression (Figure 4c). WB analysis confirmed the findings from RT-qPCR (Figure 4d and Figure S2), further validating the targeted interaction between *SMAD4* and *miR-370-3p*.

### 3.4. Detection of SMAD4 siRNA and Overexpression Plasmid Transfection Efficiency

**Figure 5:**
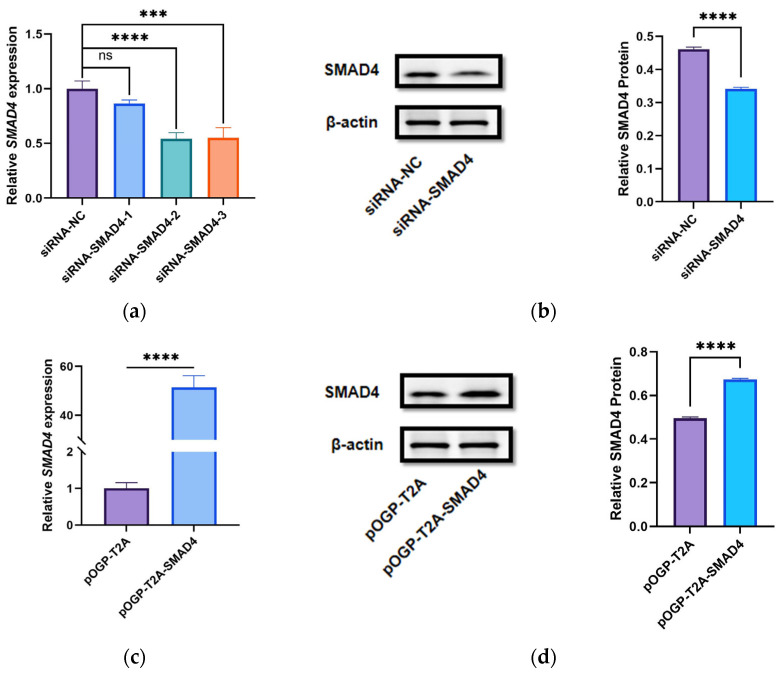
*SMAD4* siRNA sequence screening and overexpression plasmid verification. (**a**) Screening of siRNA sequences. (**b**) Effect of siRNA-*SMAD4* on *SMAD4* protein levels; Gray value analysis. (**c**) Effect of pOGP-T2A-*SMAD4* on *SMAD4* mRNA levels. (**d**) Effect of pOGP-T2A-*SMAD4* on *SMAD4* protein levels; Gray value analysis. The data are shown as mean ± SD (n = 3). Statistical significance was established with *** *p* < 0.001 and **** *p* < 0.0001, whilst “ns” denotes no significant difference.

We transfected siRNA-NC, siRNA-*SMAD4*-1, siRNA-*SMAD4*-2, and siRNA-*SMAD4*-3 into DPCs for 48 h, followed by RT-qPCR to assess the knockdown efficiency of the siRNAs. Results indicated that siRNA-*SMAD4*-2 exhibited the most effective knockdown (Figure 5a) and was designated as siRNA-*SMAD4* for subsequent experiments. Further validation using WB confirmed significant interference by siRNA-*SMAD4* on *SMAD4* protein expression levels (Figure 5b and Figure S3). Overall, these results demonstrated successful knockdown of *SMAD4* mRNA and protein levels by siRNA-*SMAD4*.

Next, we transfected the constructed overexpression plasmid pOGP-T2A-*SMAD4* into DPCs for 48 h, followed by RT-qPCR and WB to assess the overexpression efficiency of the plasmid. The findings demonstrated that pOGP-T2A-*SMAD4* markedly enhanced *SMAD4* expression both at the mRNA and protein levels, validating its suitability for further experimental protocols (Figure 5b,d and Figure S4).

### 3.5. miR-370-3p Modulates Cellular Phenotype Through SMAD4

**Figure 6:**
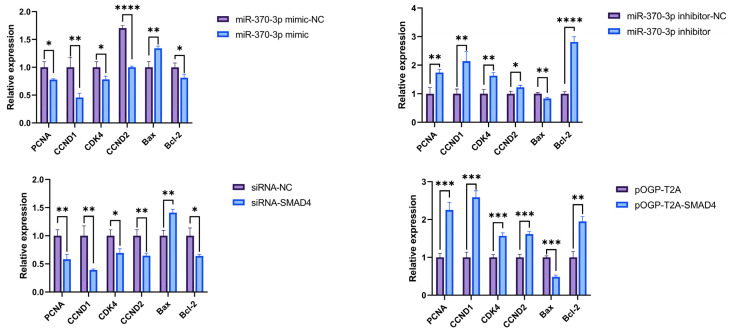
The impact of *miR-370-3p* and *SMAD4* on the mRNA expression of genes related to cellular phenotypic traits. Data are expressed as mean ± SD (n = 3), * *p* < 0.05, ** *p* < 0.01, *** *p* < 0.001, **** *p* < 0.0001.

This study investigated the impact of *miR-370-3p* and *SMAD4* on the mRNA level of genes associated with cell phenotype (Figure 6). The findings indicated that *miR-370-3p* substantially reduced the expression of the proliferation-related genes Proliferating Cell Nuclear Antigen (*PCNA*) and *CCND1* mRNA; considerably diminished the presentation of the cell cycle-associated genes Cyclin-dependent kinase 4 (*CDK4*) and Cyclin D2 (*CCND2*) mRNA; significantly enhanced the mRNA expression of the apoptosis-related gene BCL2-associated X protein (*Bax*) and suppressed B-cell lymphoma 2 (*Bcl-2*) mRNA expression. The results indicated that *miR-370-3p* might inhibit the proliferation of DPCs and affect the cell cycle.

Transfection of siRNA-NC, siRNA-*SMAD4*, pOGP-T2A, and pOGP-T2A-*SMAD4* into DPCs for 48 h, followed by RT-qPCR, revealed that *SMAD4* significantly upregulated the expression of proliferation-related genes *PCNA* and *CCND1* mRNA; substantially increased the expression of cell cycle-associated genes *CDK4* and *CCND2* mRNA; significantly downregulated the expression of the apoptosis-related gene *Bax* mRNA; and markedly increased *Bcl-2* mRNA expression. The results indicated that *SMAD4* might promote the proliferation of DPCs and affect the cell cycle.

**Figure 7:**
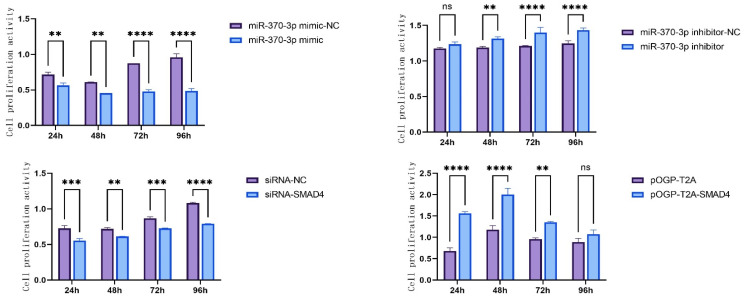
The impact of *miR-370-3p* and *SMAD4* on cellular proliferation. Data are expressed as mean ± SD (n = 3), ** *p* < 0.01, *** *p* < 0.001, **** *p* < 0.0001, ns: no significance.

This research examined the influence of *miR-370-3p* and *SMAD4* on DPC proliferation by the CCK-8 test. The findings indicated that the overexpression of *miR-370-3p* significantly inhibited DPC proliferation consistently from 24 to 96 h. The suppression of *miR-370-3p* expression did not notably affect proliferation during the 0–24 h interval but dramatically enhanced proliferation from 48 to 96 h. Transfection of siRNA-NC, siRNA-*SMAD4*, pOGP-T2A, and pOGP-T2A-*SMAD4* into DPCs for 48 h, followed by CCK-8 tests, demonstrated that *SMAD4* overexpression markedly enhanced cell proliferation from 24 to 96 h. Conversely, interference with *SMAD4* expression significantly inhibited cell proliferation from 24 to 72 h, with no significant difference observed at 96 h. Overall, the study results demonstrate that *miR-370-3p* targets *SMAD4* to suppress DPC proliferation (Figure 7).

**Figure 8:**
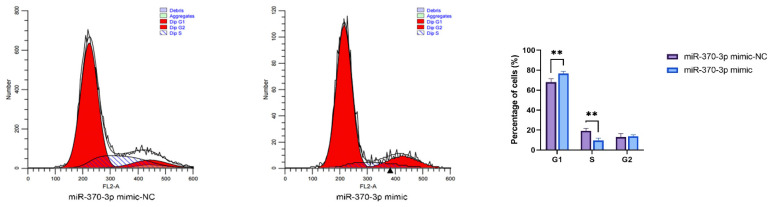

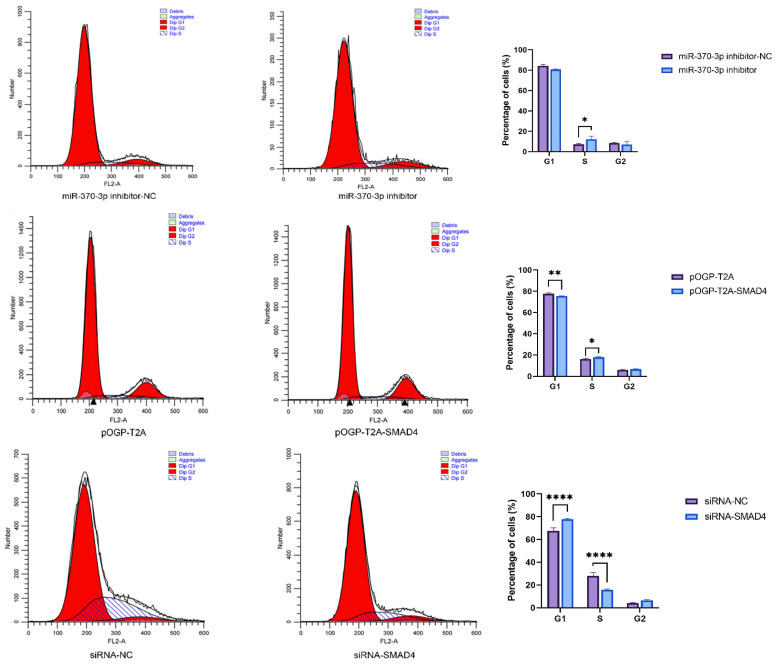
Flow cytometric examination of cell cycle distribution, encompassing statistical assessment of the proportions of cells in the G1, S, and G2 phases, based on the flow cytometry data displayed on the left. Data are expressed as mean ± SD (n = 3), * *p* < 0.05, ** *p* < 0.01, **** *p* < 0.0001.

Cell cycle analysis revealed that the overexpression of *miR-370-3p* led to a substantial buildup of cells in the G1 phase and a marked reduction in the proportion of cells in the S phase compared to the control group. Suppression of *miR-370-3p* expression significantly increased the quantity of cells in the S phase compared to the controls. Transfection of siRNA-NC, siRNA-*SMAD4*, pOGP-T2A, and pOGP-T2A-*SMAD4* into DPCs for 48 h followed by cell cycle analysis showed that interference with *SMAD4* expression markedly elevated the number of cells in the G1 phase while reducing the number of cells in the S phase relative to the control group, with no substantial change noted in the G2 phase. Conversely, *SMAD4* overexpression significantly decreased the cell count in the G1 phase and increased the cell count in the S phase compared to the control group, with no significant alteration observed in the G2 phase. These data imply that *miR-370-3p* targeting *SMAD4* plays a regulatory function in the cell cycle of DPCs (Figure 8).

**Figure 9:**
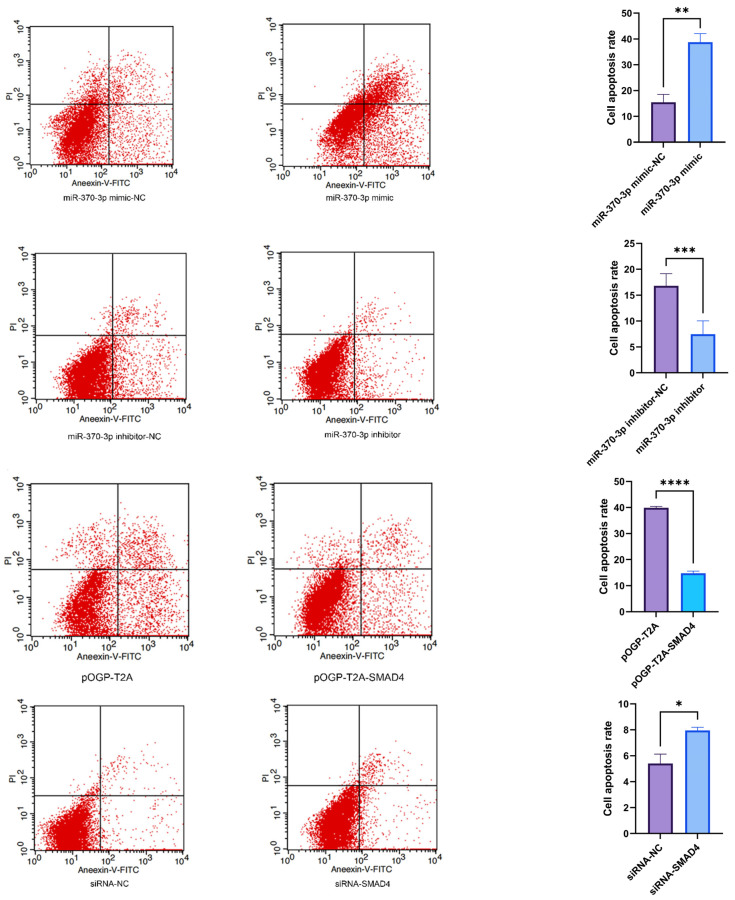
Flow cytometric examination of apoptosis and the computation of apoptosis rate, ascertained by evaluating the fraction of apoptotic cells from the left flow cytometry data. Data are expressed as mean ± SD (n = 3), * *p* < 0.05, ** *p* < 0.01, *** *p* < 0.001, **** *p* < 0.0001.

Furthermore, to assess the impact of *miR-370-3p* on DPC apoptosis, Annexin V-FITC/PI dual labeling was performed. The results demonstrated that the overexpression of *miR-370-3p* significantly increased the apoptosis rate compared to the control group, whereas the inhibition of *miR-370-3p* expression substantially decreased the apoptosis rate. The transfer of siRNA-NC, siRNA-*SMAD4*, pOGP-T2A, and pOGP-T2A-*SMAD4* into DPCs for 48 h, followed by Annexin V-FITC/PI staining, demonstrated that the downregulation of *SMAD4* expression markedly increased the apoptosis rate compared to the control group, while *SMAD4* overexpression significantly decreased apoptosis relative to the control. The findings demonstrate that *miR-370-3p* induces apoptosis in dermal DPCs via targeting *SMAD4* (Figure 9).

### 3.6. miR-370-3p Binds SMAD4 and Modulates Gene Expression in DPCs

The upstream and downstream genes of *SMAD4* within the Wnt/β-catenin signaling cascade were analyzed after transfection of DPCs with *miR-370-3p* mimic-NC, *miR-370-3p* mimic, *miR-370-3p* inhibitor-NC, *miR-370-3p* inhibitor, siRNA-NC, siRNA-*SMAD4*, pOGP-T2A, and pOGP-T2A-*SMAD4* for 48 h. RT-qPCR was used to detect the mRNA expression levels of *JUN*, *c-MYC*, transcription factor 7-like 1 (*TCF7L1*), transcription factor 7-like 2 (*TCF7L2*), and *β-catenin* in DPCs.

**Figure 10:**
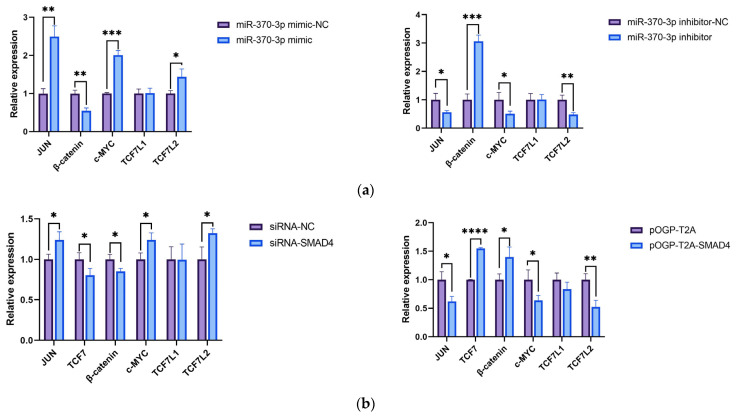
(**a**) The impact of *miR-370-3p* on the mRNA expression levels of critical genes associated with the Wnt/β-catenin signaling pathway. (**b**) Impact of *SMAD4* on the mRNA expression of genes associated with the Wnt/β-catenin signaling pathway. Data are expressed as mean ± SD (n = 3), * *p* < 0.05, ** *p* < 0.01, *** *p* < 0.001, **** *p* < 0.0001.

When *miR-370-3p* was overexpressed, there was a significant increase in *JUN*, *c-MYC*, and *TCF7L2* expression, and a marked inhibition of *β-catenin* expression, with no significant effect on *TCF7L1* expression (Figure 10a). Conversely, inhibition of *miR-370-3p* expression significantly suppressed *JUN*, *c-MYC*, and *TCF7L2* expression, markedly promoted *β-catenin* expression, and showed no significant effect on *TCF7L1* expression.

Furthermore, upon interference of *SMAD4* expression, there was significant suppression of transcription factor 7 (*TCF7*) and *β-catenin* expression, and significant promotion of *JUN*, *c-MYC*, and *TCF7L2* expression, with no significant effect on *TCF7L1* expression (Figure 10b). Also, overexpression of *SMAD4* significantly promoted *TCF7* and *β-catenin* expression, suppressed *JUN*, *c-MYC*, and *TCF7L2* expression, and had no discernible impact on the expression of *TCF7L1*.

**Figure 11:**
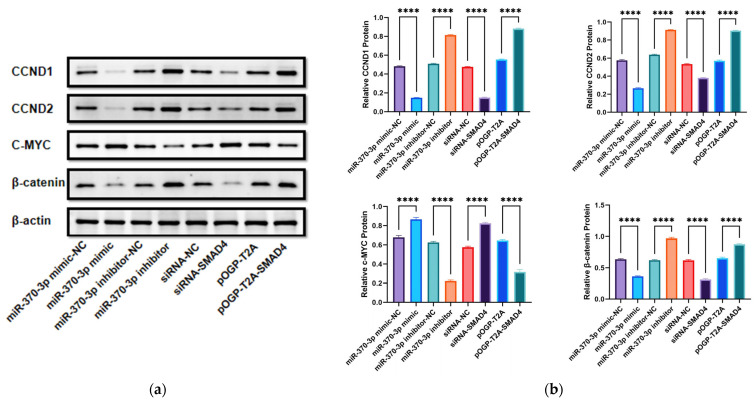
(**a**): Impact of *miR-370-3p* and *SMAD4* on the protein expression levels of genes within the DPCs. (**b**): Analysis of protein grayscale. Data are expressed as mean ± SD (n = 3), **** *p* < 0.0001.

In addition, following the transfection of *miR-370-3p* mimic-NC, *miR-370-3p* mimic, *miR-370-3p* inhibitor-NC, *miR-370-3p* inhibitor, siRNA-NC, siRNA-*SMAD4*, pOGP-T2A, and pOGP-T2A-*SMAD4* into DPCs for 48 h, the protein expression levels of CCND1, CCND2, c-MYC, and *β*-catenin were evaluated by Western blotting. The results showed that *miR-370-3p* overexpression significantly increased c-MYC expression while significantly reducing *β*-catenin, CCND1, and CCND2 expression (Figure 11a,b and Figure S4). CCND1, CCND2, and *β*-catenin expression were significantly increased while c-MYC expression was significantly decreased when *miR-370-3p* expression was inhibited. Moreover, interference with *SMAD4* expression significantly suppressed CCND1, CCND2, and *β*-catenin expression, while significantly promoting c-MYC expression. However, overexpression of *SMAD4* significantly suppressed c-MYC expression and significantly promoted CCND1, CCND2, and *β*-catenin expression.

## 4. Discussion

Numerous cell types, including DPCs and hair follicle stem cells (HFSCs), carefully regulate the growth and development of hair follicles. HFSCs are vital for sustaining the long-term regeneration potential of hair follicles, whereas DPCs deliver crucial signals that direct follicular morphogenesis, cycling, and differentiation. Together, these cell populations interact within a highly coordinated microenvironment to ensure proper follicular function and hair production. Wnt/*β*-catenin, Notch, Sonic Hedgehog (Shh), and BMP are among the several signaling pathways that control the growth and differentiation of hair follicles. These pathways control key processes like cell proliferation and differentiation, ensuring proper hair follicle function [13]. The genetic regulatory network governing hair follicle development is highly complex, encompassing a range of signaling pathways, including Wnt, BMP, EDAR, and Shh. These pathways function interactively within the follicular microenvironment, coordinating cellular processes that regulate follicle morphogenesis and cycling [2,14]. MicroRNAs influence the biological processes of cancer, including invasion, metastasis, apoptosis, and proliferation, by controlling significant signaling pathways such as PI3K/Akt, MAPK, and Wnt [15].

MicroRNAs (miRNAs) are diminutive, non-coding RNA molecules, typically around 24 nucleotides in length, that regulate gene expression post-transcriptionally. They affect outcomes by binding to specific areas in the 3′UTR of target mRNAs, leading to either mRNA destruction or translational repression. MiRNAs have a vital role in regulating multiple biological processes, including cell differentiation, proliferation, and apoptosis, through these pathways [16]. They provide crucial regulatory functions in organismal growth and development, and research has underscored the important functions of *miR-370-3p* in disorders like myocardial infarction, heart failure, lung cancer of non-small cells, and pneumonia [17,18,19]. Moreover, *miR-370-3p* has been linked to the regulation of tumors and malignancies. Jia et al. demonstrated that *CircCCNB1* suppresses cervical cancer proliferation through the *miR-370-3p*/*SOX4* pathway, emphasizing the significance of circular RNAs (circRNAs) in the regulation of cancer-related signaling pathways [20]. This study highlights the crucial function of *miR-370-3p* in facilitating the regulatory impact of *circCCNB1* on carcinogenic mechanisms. Additionally, it has been suggested that *circ_0025033* facilitates the advancement of ovarian cancer by modulating the has-*miR-370-3p*/*SLC1A5* pathway. This underscores the regulatory role of circular RNAs (circRNAs) in influencing miRNA activity, which in turn affects critical cellular processes such as amino acid transport. Such regulation may be integral to tumor growth and metabolism, thus highlighting the complex molecular interactions contributing to ovarian cancer progression [21]. Experimental evidence by Li et al. confirmed that *circARID1A* regulated glioblastoma migration and invasion via the *miR-370-3p*/*TGFBR2* axis [22]. Additionally, *miR-370-3p* is implicated in the control of several physiological processes in rats, and studies have indicated its role in the regulation of goat hair follicle development. Considering the notable disparities in wool fiber diameter between Xinjiang fine-wool sheep and Tan sheep, together with prior research on *miR-370-3p* in goats conducted by Haierhan, it has been deduced that *miR-370-3p* may be linked to follicles for hair growth and development. This study gathered skin samples from sheep in October (hair follicle growth phase) and January (hair follicle resting phase) for fluorescence quantitative PCR, revealing significant differences in spatiotemporal expression of *miR-370-3p* in skin tissues. This suggests that *miR-370-3p* may have a regulatory role in the growth, cycle, and phenotype of sheep hair follicles. The analysis of *miR-370-3p* tissue expression demonstrated significant variation in expression patterns between the two sheep breeds. Target gene validation using dual-luciferase reporter assays and detection at the gene and protein levels confirmed *SMAD4* as a target for *miR-370-3p*, consistent with previous studies. Subsequent transient transfection assays on DPCs confirmed the targeting efficacy of *miR-370-3p* on *SMAD4*, leading to the downregulation of *SMAD4* expression.

Prior studies have highlighted the pivotal function of the SMAD family in regulating hair follicle cycling and the differentiation of associated cells, hence indicating its significance in the molecular pathways governing hair follicle growth and homeostasis. *SMAD2* has been suggested to modulate hair follicle development and growth in Angora rabbit dermal tissues through the TGF-*β* signaling pathway, underscoring the critical role of SMAD family members in hair follicle dynamics [23]. Research demonstrates that *miR-203a-3p* inhibits *SMAD1* expression, hence facilitating the process of differentiation of HFSCs induced by loureirin A. This shows that *miR-203a-3p* is pivotal in regulating HFSC differentiation by modulating the *SMAD1* signaling pathway [12]. Nan et al. [24] revealed that All-trans-retinoic acid (*ATRA*) reduces DPC proliferation and promotes apoptosis via the TGF-β2/Smad2/3 signaling pathway, therefore decreasing hair follicle development. This finding underscores the regulatory influence of *ATRA* on hair follicle dynamics via modulation of essential biological mechanisms, including growth and apoptosis. *SMAD7* is acknowledged as an antagonist of Wnt/β-catenin signaling, and its activation can alter the differentiation of skin cells from hair follicle production to sebaceous gland development. This indicates that *SMAD7* is essential in regulating the equilibrium between hair follicle and sebaceous gland development by opposing the Wnt/β-catenin pathway, therefore affecting skin appendage differentiation [25]. Li et al. proposed that downregulation of the transcription factor *SMAD3* inhibits TGF-β signaling, inducing premature entry and prolongation of dormant hair follicles into the growth phase [26]. These findings imply that the *SMAD4* protein may influence the hair follicle cycle and associated cellular phenotypes, thereby contributing to variations in wool traits. This suggests that *SMAD4* may regulate critical processes, including follicular differentiation, growth, and development, which are vital for defining wool characteristics. Additional study is necessary to clarify the specific molecular pathways by which *SMAD4* influences hair follicle dynamics and its possible impact on wool phenotypes. Research has demonstrated that the ablation of the *SMAD4* gene leads to the deterioration of hair follicles in the epidermis, highlighting its essential role in regulating hair follicle growth and development [10,27]. *SMAD4* is essential for the proper development of hair follicles, and its functional deletion in mice disrupts the normal hair follicle cycle. This disturbance can result in abnormal skin development, thereby elevating the risk of skin cancers or carcinomas [27,28].

Park et al. suggested that the *Wip1* gene inhibits TGF-β signaling by regulating *SMAD4* phosphorylation [29]. Wang et al. demonstrated that the inhibition of *TGF-β1* gene expression leads to reduced *SMAD4* phosphorylation, hence promoting apoptosis and impeding proliferation of cells, assault, and the transition from epithelial to mesenchymal in gallbladder cancer. The results highlight the crucial role of the TGF-β/*SMAD4* signaling pathway in regulating vital cellular processes in cancer growth and suggest potential therapeutic options for targeting this pathway in gallbladder cancer [30]. Structural analyses reveal that the MH1 and MH2 modules of *SMAD4* are linked by a flexible region, enabling dynamic conformational changes. This flexibility is crucial for the protein’s function, as it facilitates transient interactions with other SMAD proteins and transcriptional regulators, thereby modulating its role in TGF-β signaling and cellular responses [31]. These results indicate that *SMAD4* may modulate cell phenotypes by affecting cellular processes vital for the organism’s physiological functioning, such as proliferation, differentiation, and death. This regulation likely contributes to tissue development and overall homeostasis.

To elucidate the regulatory function of the *SMAD4* gene in sheep, real-time quantitative PCR (qPCR) was utilized to assess the expression levels of *SMAD4* across different tissues in Small-tail Han sheep and Xinji fine-wool sheep. We found that there were significant differences in heart, spleen, skin, lung, and muscle tissues between the two breeds. Considering the substantial disparities in wool fiber diameter and hair follicle density between Small-tail Han sheep and Xinji fine-wool sheep, together with the pronounced variations in *SMAD4* gene expression in the skin tissues of both breeds in January (hair follicle growth phase), We hypothesize that the *SMAD4* gene may be involved in the regulation of hair follicle growth and development, aligning with predictions derived from protein interaction analysis. Immunohistochemistry results further validated the expression of the *SMAD4* protein in multiple parts of the hair follicle, confirming our hypothesis. This study validated the expression of the *SMAD4* gene in several sheep tissues, underscoring its essential role in hair follicle growth and development. Future research will employ DPCs from this study to analyze the regulatory effects of *miR-370-3p* and *SMAD4* genes on cell phenotypes and the mechanisms governing hair follicle cell cycles in Tan sheep.

Through RT-qPCR, cell phenotype-related genes (*PCNA*, *CCND1*, *CDK4*, *CCND2*, *Bax*, *Bcl-2*) were preliminarily determined to be affected by *miR-370-3p* in DPCs, influencing cell proliferation, cycle, and apoptosis, possibly through targeting *SMAD4*. This study utilized CCK-8 assays and flow cytometry to substantiate these hypotheses, demonstrating that *miR-370-3p* inhibits DPC proliferation and affects the cell cycle, perhaps by regulating related genes, consistent with previous research [32]. Research using Annexin V-FITC and PI dual staining demonstrated that *miR-370-3p* promotes cellular death. In contrast, *SMAD4* affects DPC phenotype differently; it promotes DPC proliferation, inhibits apoptosis, and has a certain effect on the cell cycle. Overall, it is inferred that *miR-370-3p* participates in regulating DPC cell phenotypes by targeting *SMAD4*.

Currently, there is no relevant research on the effects of *miR-370-3p* and *SMAD4* on sheep dermal papilla cells (DPCs). In this study, to explore the molecular mechanism by which *miR-370-3p* regulates *SMAD4* and affects the development and growth of DPCs, the expression levels of relevant genes in DPCs were detected. The results showed that the targeting of *SMAD4* by *miR-370-3p* could affect the expression of relevant genes such as *JUN*, *c-MYC*, and *β-catenin*. To further validate the results of real-time quantitative polymerase chain reaction (RT-qPCR), we selected several key proteins (cyclin D1 (CCND1), cyclin D2 (CCND2), β-catenin, and c-MYC) for Western blot analysis. The results were consistent with those of RT-qPCR. I would argue that *SMAD4* participates in several biological processes in cross-talk with the Wnt/β-catenin pathway [33,34,35,36]. Regarding the phenomenon that *SMAD4* affects the expression of the upstream gene β-catenin, we speculated that there might be a negative regulatory mechanism, which needs to be verified in the next stage of our research.

## 5. Conclusions

In this study, we utilized RT-qPCR to validate the expression profiles of *miR-370-3p* and *SMAD4* in various tissues of two sheep breeds. A luciferase reporter assay confirmed the targeting relationship between *miR-370-3p* and *SMAD4*. Immunohistochemistry and PCR verified the expression of *SMAD4* in dermal papilla cells (DPCs). Combined with the CCK8 assay and flow cytometry, we examined the cells transfected with plasmids. The results indicated that *miR-370-3p* inhibited the proliferation of DPCs, whereas *SMAD4* promoted DPC proliferation, and both had an impact on the cell cycle. Finally, we employed a real-time quantitative polymerase chain reaction (RT-qPCR) and Western blot to explore the expression of relevant genes in DPCs. The results showed that *miR-370-3p* influenced the expression of genes such as JUN, c-MYC, and β-catenin. Therefore, *miR-370-3p* participates in regulating the proliferation, cell cycle progression, and apoptosis of DPCs by targeting and regulating the expression of *SMAD4*.

## Figures and Tables

**Table 1:** Primers used for qRT-PCR.

Primer Name	Primer Sequence (from 5′ to 3′)	Usage
*miR-370-3p*	TGCTGGGGTGGAACCTGGT	miRNA qRT-PCR
*SMAD4*-F	TGAGCTTGCATTCCAGCCTCC	*SMAD4* qRT-PCR
*SMAD4*-R	CCAAGCAAAAGCGATCTCCTCC	*SMAD4* qRT-PCR
β-actin-F	CCGCAAATGCTTCTAGGCGG	β-actin qRT-PCR
β-actin-R	TCGCACGAGGCCAATCTCAT	β-actin qRT-PCR
PCNA-F	TGGGACATCAGCTCAAGTGG	PCNA qRT-PCR
PCNA-R	AAGGGTTAGCTGCACCAAGG	PCNA qRT-PCR
CCND1-F	CCTCTCCTATCACCGCCTGA	CCND1 qRT-PCR
CCND1-R	TTTGGGGTCCAAGTTCTGCT	CCND1 qRT-PCR
CDK4-F	ACCTCTCGATACGAGCCAGT	CDK4 qRT-PCR
CDK4-R	CGTTGGGGACTCTCACACTC	CDK4 qRT-PCR
CCND2-F	AGCACGCTCAGACCTTCATC	CCND2 qRT-PCR
CCND2-R	AGGCAATCCACATCCGTGTT	CCND2 qRT-PCR
Bcl-2-F	TGAGTTCGGAGGGGTCATGT	Bcl-2 qRT-PCR
Bcl-2-R	GGTACTCGGTCATCCACAGG	Bcl-2 qRT-PCR
Bax-F	TGTCGCCCTTTTCTACTTTGCC	Bax qRT-PCR
Bax-R	AATGTCCAGCCCATGATGGTC	Bax qRT-PCR
c-MYC-F	CCCCTGCCAAAAGGTCAGAATCGG	c-MYC qRT-PCR
c-MYC-R	ACGTGGCATCTCTTTAGGACCA	c-MYC qRT-PCR
β-catenin-F	TCAGGATACCCAGCGTCGTA	β-catenin qRT-PCR
β-catenin-R	AGCAGCTGCACAAACAATGG	β-catenin qRT-PCR
JUN-F	GCTTCCAAGTGCCGGAAAAG	JUN qRT-PCR
JUN-R	GCTGCGTTAGCATGAGTTGG	JUN qRT-PCR
TCF7-F	GAAAAGCACCAAGAATCCAAC	TCF7 qRT-PCR
TCF7-R	CTAGAGCACTGTCATCGGAA	TCF7 qRT-PCR
TCF7L1-F	GCAAATCCCACATCCCCTCA	TCF7L1 qRT-PCR
TCF7L1-R	CACCATGTGAGGGGAGAACC	TCF7L1 qRT-PCR
TCF7L2-F	ACCTGTCCATGATGCCTCCG	TCF7L2 qRT-PCR
TCF7L2-R	AGGAAGATGTCGACGGCTGTG	TCF7L2 qRT-PCR

**Table 2:** Information on Mutation Sites of the Vector.

Carrier Name	Mutated Site Sequence
pmirglo-oar-*miR-370-3p*-*SMAD4*-WT	6101 CCTGAAGTCAGAGGAGTCATGCCATAACTCAAGAGACGAGCCACACTTAG 6151 CTTCTGCTTT GGGGAAAACT GGTCAGCTATGG***GCTCTGGT AGGT***CCTTTG 6201 TGGCTTTCTG TATGCTTTTG CCTGGTTGAA GTCTGTGGCT AAAAAAACAG
pmirglo-oar-*miR-370-3p*-*SMAD4*-Mut	6101 CCTGAAGTCA GAGGAGTCAT GCCATAACTC AAGAGACGAG CCACACTTAG 6151 CTTCTGCTTT GGGGAAAACT GGTCAGCTAT GG***GATATGAT CTTT***CCTTTG 6201 TGGCTTTCTG TATGCTTTTG CCTGGTTGAA GTCTGTGGCT AAAAAAACAG

pmirGLO: carrier name; OAR: Ovis aries.

**Table 3:** Sequences of *miR-370-3p* mimic, *miR-370-3p* inhibitor, and siRNA-*SMAD4* used in this study.

Name	Sequences
*miR-370-3p* mimic	sense:5′-GCCUGCUGGGGUGGAACCUGGUCU-3’ anti-sense:5’-ACCAGGUUCCACCCCAGCAGGCUU-3’
*miR-370-3p* inhibitor	sense:5’-AGACCAGGUUCCACCCCAGCAGGC-3’
siRNA-*SMAD4*-1	sense:5’-GCAGCCAUAGUGAAGGAUUTT-3’ anti-sense:5’-AAUCCUUCACUAUGGCUGCTT-3’
siRNA-*SMAD4*-2	sense:5’-GCCUCCUAUUUCUAAUCAUTT-3’ anti-sense:5’-AUGAUUAGAAAUAGGAGGCTT-3’
siRNA-*SMAD4*-3	sense:5’-CCUUCACACCAUGCCUAUUTT-3’ anti-sense:5’-AAUAGGCAUGGUGUGAAGGTT-3’

## Data Availability

All datasets utilized and/or examined in this investigation are accessible from the relevant author upon reasonable request.

## Supplementary Materials

The following supporting information can be downloaded at https://www.mdpi.com/article/10.3390/cells14100714/s1; Figure S1: Expression of *SMAD4* in DPCs; Figure S2: Uncropped blots for the experiment shown in Figure 4d describing the impact of *miR-370-3p* on the expression levels of *SMAD4* and β-actin protein; Figure S3: Uncropped blots for the experiment shown in Figure 5b,d describing the impact of *SMAD4* on the expression levels of *SMAD4* and β-actin protein; Figure S4: Uncropped blots for the experiment shown in Figure 11a describing the expression levels of CCND1, CCND2,C-MYC, β-catenin, β-actin protein.

## Abbreviations

3′-UTRs	3′-untranslated regions
ANOVA	Analysis of variance
miRNAs	microRNAs
PBS	Phosphate-buffered saline
PVDF	Polyvinylidene difluoride
RT-qPCR	Quantitative real-time PCR
SDS-PAGE	Sodium dodecyl sulphate-polyacrylamide gel electrophoresis

## References

[1] Peterson A., Nair L.S. (2022). Hair Follicle Stem Cells for Tissue Regeneration. Tissue Eng. Part B Rev..

[2] Lin X., Zhu L., He J. (2022). Morphogenesis, Growth Cycle and Molecular Regulation of Hair Follicles. Front. Cell Dev. Biol..

[3] Mardaryev A.N., Ahmed M.I., Vlahov N.V., Fessing M.Y., Gill J.H., Sharov A.A., Botchkareva N.V. (2010). Micro-RNA-31 controls hair cycle-associated changes in gene expression programs of the skin and hair follicle. FASEB J..

[4] Bartel D.P. (2009). MicroRNAs: Target recognition and regulatory functions. Cell.

[5] Yan S., Jiao K. (2016). Functions of miRNAs during Mammalian Heart Development. Int. J. Mol. Sci..

[6] Cao L., Tian T., Huang Y., Tao S., Zhu X., Yang M., Gu J., Feng G., Ma Y., Xia R. (2021). Neural progenitor cell-derived nanovesicles promote hair follicle growth via miR-100. J. Nanobiotechnology.

[7] Zhao B., Chen Y., Yang N., Chen Q., Bao Z., Liu M., Hu S., Li J., Wu X. (2019). miR-218-5p regulates skin and hair follicle development through Wnt/β-catenin signaling pathway by targeting SFRP2. J. Cell. Physiol..

[8] Calvo-Sánchez M.I., Fernández-Martos S., Carrasco E., Moreno-Bueno G., Bernabéu C., Quintanilla M., Espada J. (2019). A role for the Tgf-β/Bmp co-receptor Endoglin in the molecular oscillator that regulates the hair follicle cycle. J. Mol. Cell Biol..

[9] Owens P., Bazzi H., Engelking E., Han G., Christiano A.M., Wang X.J. (2008). *SMAD4*-dependent desmoglein-4 expression contributes to hair follicle integrity. Dev. Biol..

[10] Owens P., Han G., Li A.G., Wang X.J. (2008). The role of Smads in skin development. J. Investig. Dermatol..

[11] Yang L., Wang L., Yang X. (2009). Disruption of *SMAD4* in mouse epidermis leads to depletion of follicle stem cells. Mol. Biol. Cell.

[12] Luo Z., Dou J., Xie F., Lu J., Han Q., Zhou X., Kong J., Chen D., Liu A. (2021). miR-203a-3p promotes loureirin A-induced hair follicle stem cells differentiation by targeting Smad1. Anat. Rec. Hoboken.

[13] Li J., Zhao B., Yao S., Dai Y., Zhang X., Yang N., Bao Z., Cai J., Chen Y., Wu X. (2023). Dermal PapillaCell-Derived Exosomes Regulate Hair Follicle Stem Cell Proliferation via LEF1. Int. J. Mol. Sci..

[14] Rendl M., Lewis L., Fuchs E. (2005). Molecular dissection of mesenchymal-epithelial interactions in the hair follicle. PLoS Biol..

[15] Inui M., Martello G., Piccolo S. (2010). MicroRNA control of signal transduction. Nat. Rev. Mol. Cell Biol..

[16] Huntzinger E., Izaurralde E. (2011). Gene silencing by microRNAs: Contributions of translational repression and mRNA decay. Nat. Rev. Genet..

[17] Long X., Wang D.G., Wu Z.B., Liao Z.M., Xu J.J. (2023). Circular RNA hsa_circ_0004689 (circSWT1) promotes NSCLC progression via the *miR-370-3p*/SNAIL axis by inducing cell epithelial-mesenchymal transition (EMT). Cancer Med..

[18] Wang P., Zhang H., Zhao W., Dai N. (2021). Silencing of long non-coding RNA KCNQ1OT1 alleviates LPS-induced lung injury by regulating the *miR-370-3p*/FOXM1 axis in childhood pneumonia. BMC Pulm. Med..

[19] Yanni J., D’Souza A., Wang Y., Li N., Hansen B.J., Zakharkin S.O., Smith M., Hayward C., Whitson B.A., Mohler P.J. (2020). Silencing *miR-370-3p* rescues funny current and sinus node function in heart failure. Sci. Rep..

[20] Jia C., Chen F., Li W., Zhu X., Wang Y., Guo H., Xi H. (2023). CircCCNB1 Knockdown Blocks the Progression of Cervical Cancer by Acting as Competing Endogenous RNA in the *miR-370-3p*/SOX4 Pathway. Ann. Clin. Lab. Sci..

[21] Ma H., Qu S., Zhai Y., Yang X. (2022). circ_0025033 promotes ovarian cancer development via regulating the hsa_*miR-370-3p*/SLC1A5 axis. Cell. Mol. Biol. Lett..

[22] Li B., Chen J., Wu Y., Luo H., Ke Y. (2022). Decrease of circARID1A retards glioblastoma invasion by modulating *miR-370-3p*/ TGFBR2 pathway. Int. J. Biol. Sci..

[23] Bao Z., Zhao B., Hu S., Yang N., Liu M., Li J., Liang S., Zhou T., Chen Y., Wu X. (2021). Characterization and functional analysis of SMAD2 regulation in hair follicle cycle in Angora rabbits. Gene.

[24] Nan W., Li G., Si H., Lou Y., Wang D., Guo R., Zhang H. (2020). All-trans-retinoic acid inhibits mink hair follicle growth via inhibiting proliferation and inducing apoptosis of dermal papilla cells through TGF-β2/Smad2/3 pathway. Acta Histochem..

[25] Han G., Li A.G., Liang Y.Y., Owens P., He W., Lu S., Yoshimatsu Y., Wang D., Ten Dijke P., Lin X. (2006). Smad7-induced beta-catenin degradation alters epidermal appendage development. Dev. Cell.

[26] Li Z., Ryu S.W., Lee J., Choi K., Kim S., Choi C. (2016). Protopanaxatirol type ginsenoside Re promotes cyclic growth of hair follicles via inhibiting transforming growth factor β signaling cascades. Biochem. Biophys. Res. Commun..

[27] Yuan C., Jiao L., Yang L., Ying W., Hu Z., Liu J., Cui F., Li L., Qian L., Teng Y. (2008). The up-regulation of 14-3-3 proteins in *SMAD4* deficient epidermis and hair follicles at catagen. Proteomics.

[28] Yang L., Mao C., Teng Y., Li W., Zhang J., Cheng X., Li X., Han X., Xia Z., Deng H. (2005). Targeted disruption of *SMAD4* in mouse epidermis results in failure of hair follicle cycling and formation of skin tumors. Cancer Res..

[29] Park D.S., Yoon G.H., Kim E.Y., Lee T., Kim K., Lee P.C., Chang E.J., Choi S.C. (2020). Wip1 regulates *SMAD4* phosphorylation and inhibits TGF-β signaling. EMBO Rep..

[30] Wang Z., Zhao X., Ma Z., Liu L., Wang B., Li Y. (2018). WITHDRAWN: Modulation on gallbladder carcinoma by TGF-β1 via IGFBP-2. Cancer Biomark..

[31] Gomes T., Martin-Malpartida P., Ruiz L., Aragón E., Cordeiro T.N., Macias M.J. (2021). Conformational landscape of multidomain SMAD proteins. Comput. Struct. Biotechnol. J..

[32] Hai E., Han W., Wu Z., Ma R., Shang F., Wang M., Liang L., Rong Y., Pan J., Wang Z. (2021). Chi-*miR-370-3p* regulates hair follicle morphogenesis of Inner Mongolian cashmere goats. G3 Bethesda.

[33] Du X., Li Q., Yang L., Liu L., Cao Q., Li Q. (2020). *SMAD4* activates Wnt signaling pathway to inhibit granulosa cell apoptosis. Cell Death Dis..

[34] Lanauze C.B., Sehgal P., Hayer K., Torres-Diz M., Pippin J.A., Grant S.F.A., Thomas-Tikhonenko A. (2021). Colorectal Cancer-Associated *SMAD4* R361 Hotspot Mutations Boost Wnt/β-Catenin Signaling through Enhanced *SMAD4*-LEF1 Binding. Mol. Cancer Res..

[35] McCarthy S.S., Karolak M., Oxburgh L. (2022). *SMAD4* controls proliferation of interstitial cells in the neonatal kidney. Development.

[36] Voorneveld P.W., Kodach L.L., Jacobs R.J., van Noesel C.J., Peppelenbosch M.P., Korkmaz K.S., Molendijk I., Dekker E., Morreau H., van Pelt G.W. (2015). The BMP pathway either enhances or inhibits the Wnt pathway depending on the *SMAD4* and p53 status in CRC. Br. J. Cancer.

