# Supplementary material for: miR-370-3p Inhibited the Proliferation of Sheep Dermal Papilla Cells by Inhibiting the Expression of SMAD4
Source: Cells. 2025 May 14;14(10):714. doi: 10.3390/cells14100714 (PMC12110447; doi:10.3390/cells14100714)
Supplement: Supplementary file 1 [file cells-14-00714-s001.zip › Supplementary Figure S2.pdf]

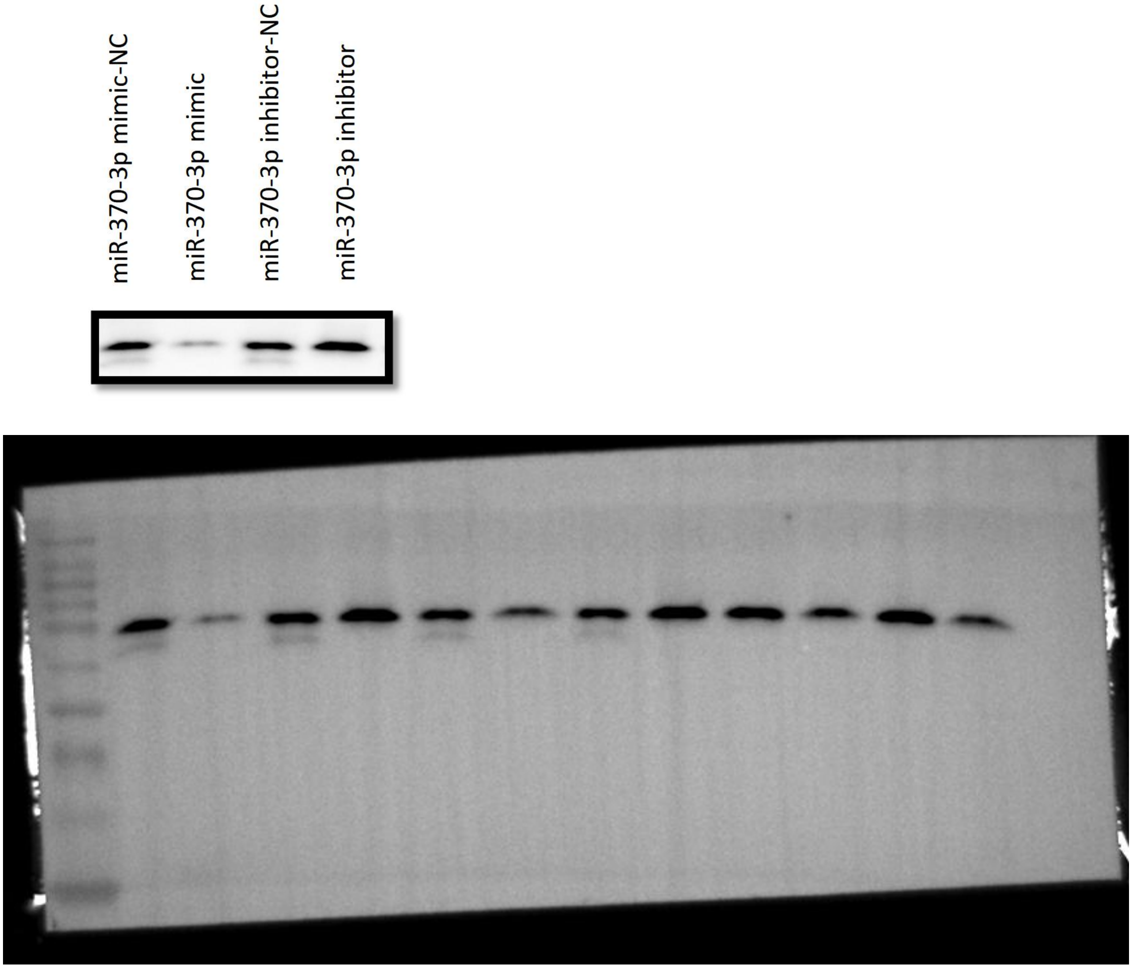
miR-370-3p mimic-NC
miR-370-3p mimic
miR-370-3p inhibitor-NC
miR-370-3p inhibitor

SMAD4

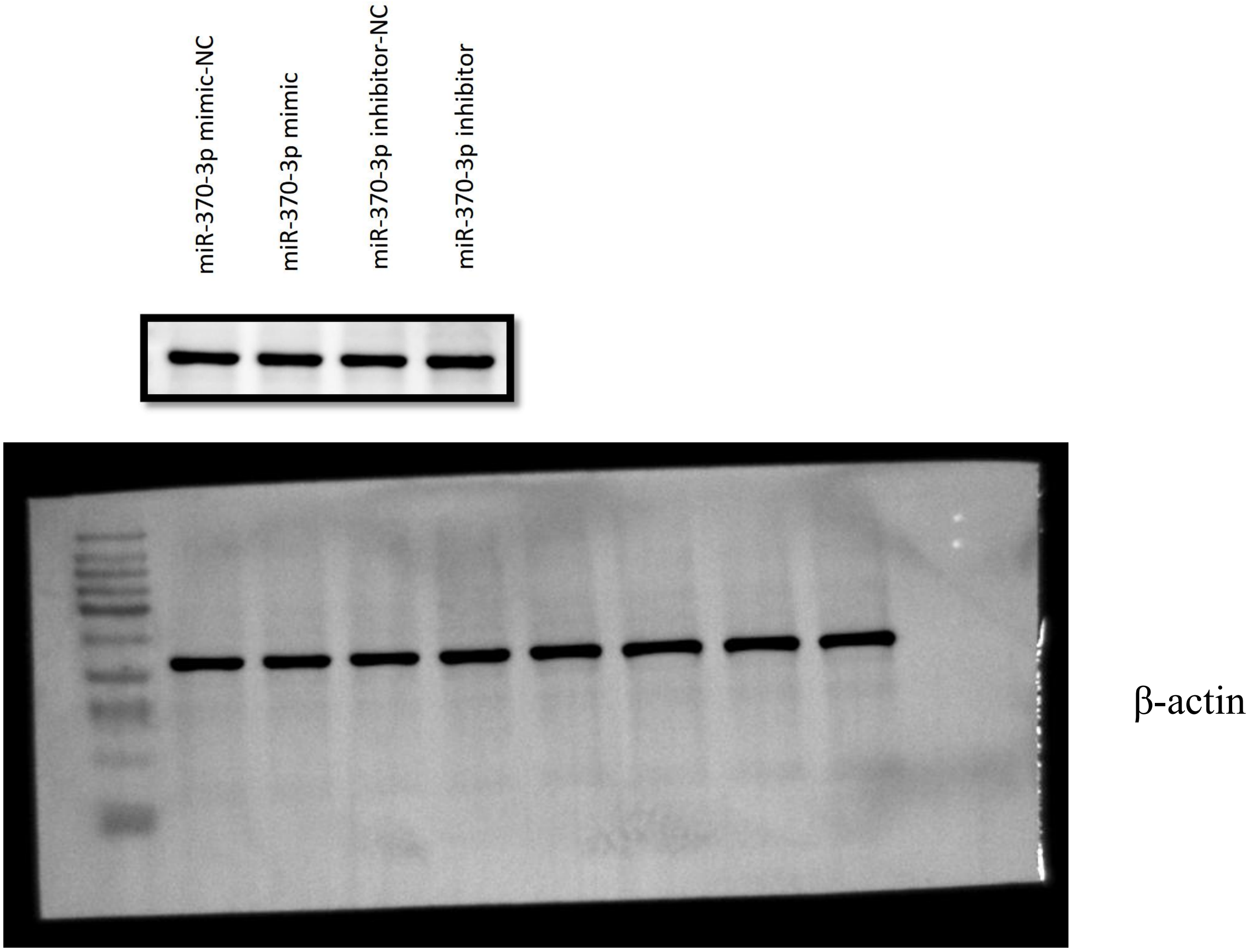

Supplementary Figure S2. Uncropped blots for the experiment shown in Figure 4d describing the impact of miR-370-3p on the expression levels of SMAD4 and β-actin protein.
