# Supplementary material for: miR-370-3p Inhibited the Proliferation of Sheep Dermal Papilla Cells by Inhibiting the Expression of SMAD4
Source: Cells. 2025 May 14;14(10):714. doi: 10.3390/cells14100714 (PMC12110447; doi:10.3390/cells14100714)
Supplement: Supplementary file 1 [file cells-14-00714-s001.zip › Supplementary Figure S3.pdf]

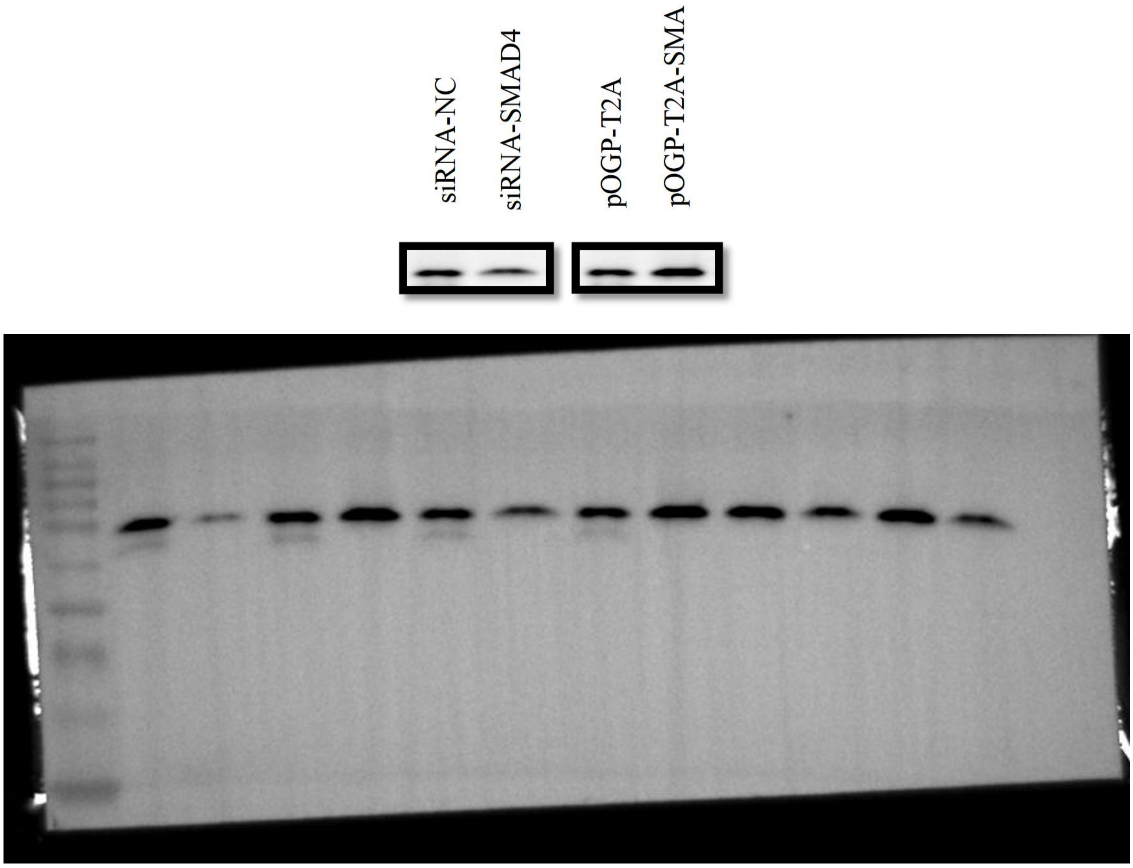

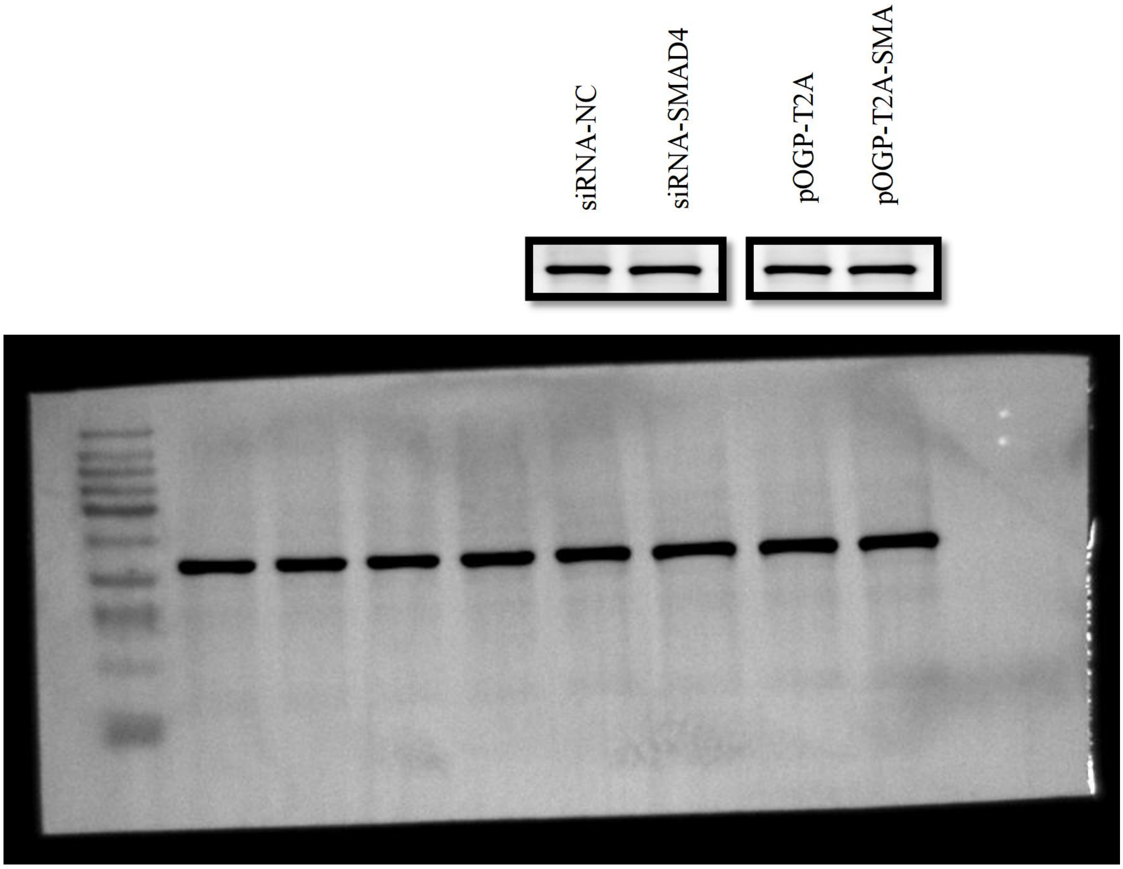

Supplementary Figure S3. Uncropped blots for the experiment shown in Figure 5b,d describing the impact of SMAD4 on the expression levels of SMAD4 and β-actin protein.
