# Supplementary material for: miR-370-3p Inhibited the Proliferation of Sheep Dermal Papilla Cells by Inhibiting the Expression of SMAD4
Source: Cells. 2025 May 14;14(10):714. doi: 10.3390/cells14100714 (PMC12110447; doi:10.3390/cells14100714)
Supplement: Supplementary file 1 [file cells-14-00714-s001.zip › Supplementary Figure S4.pdf]

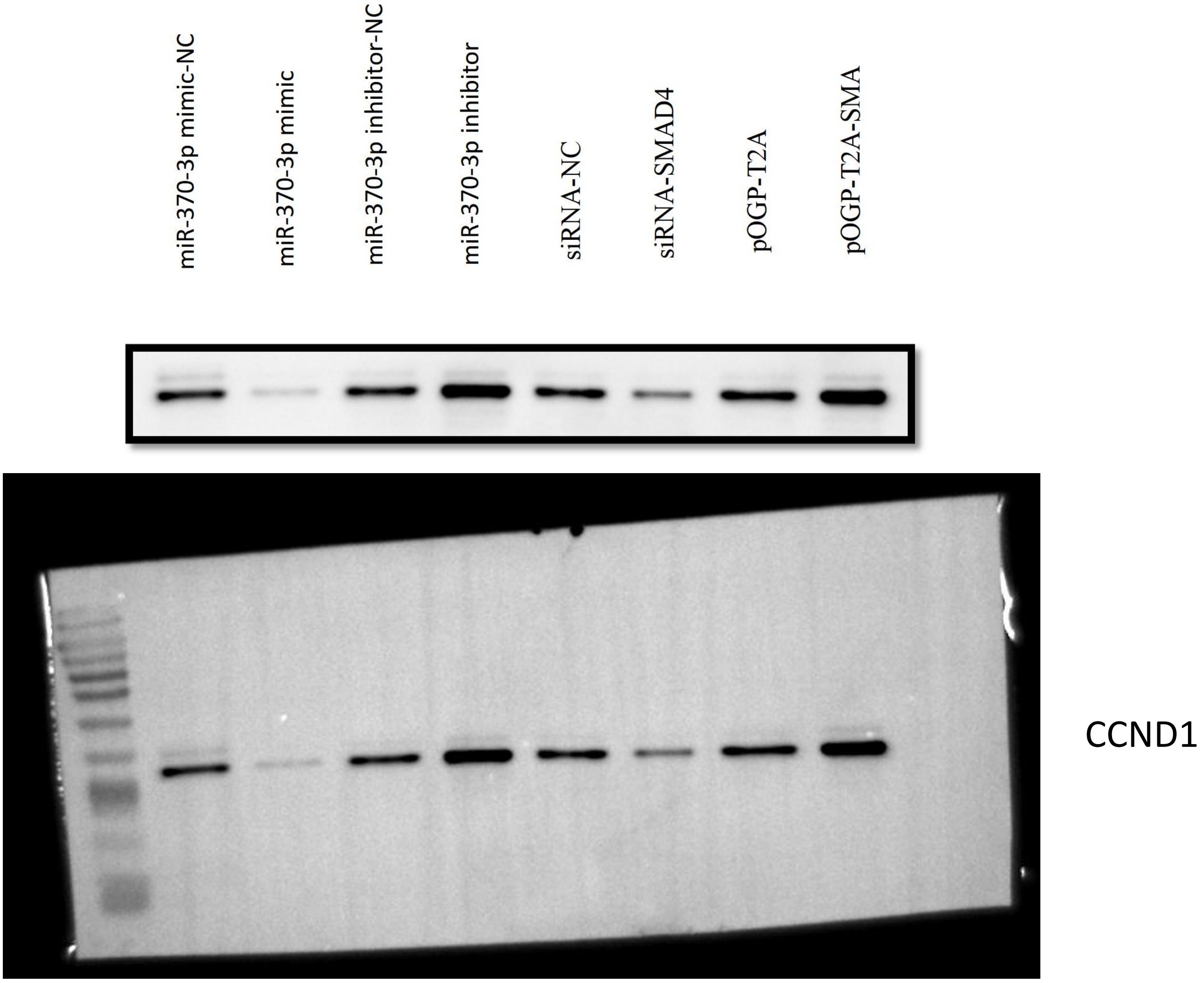
miR-370-3p mimic-NC
miR-370-3p mimic
miR-370-3p inhibitor-NC
miR-370-3p inhibitor
siRNA-NC
siRNA-SMAD4
pOGP-T2A
pOGP-T2A-SMA
CCND1

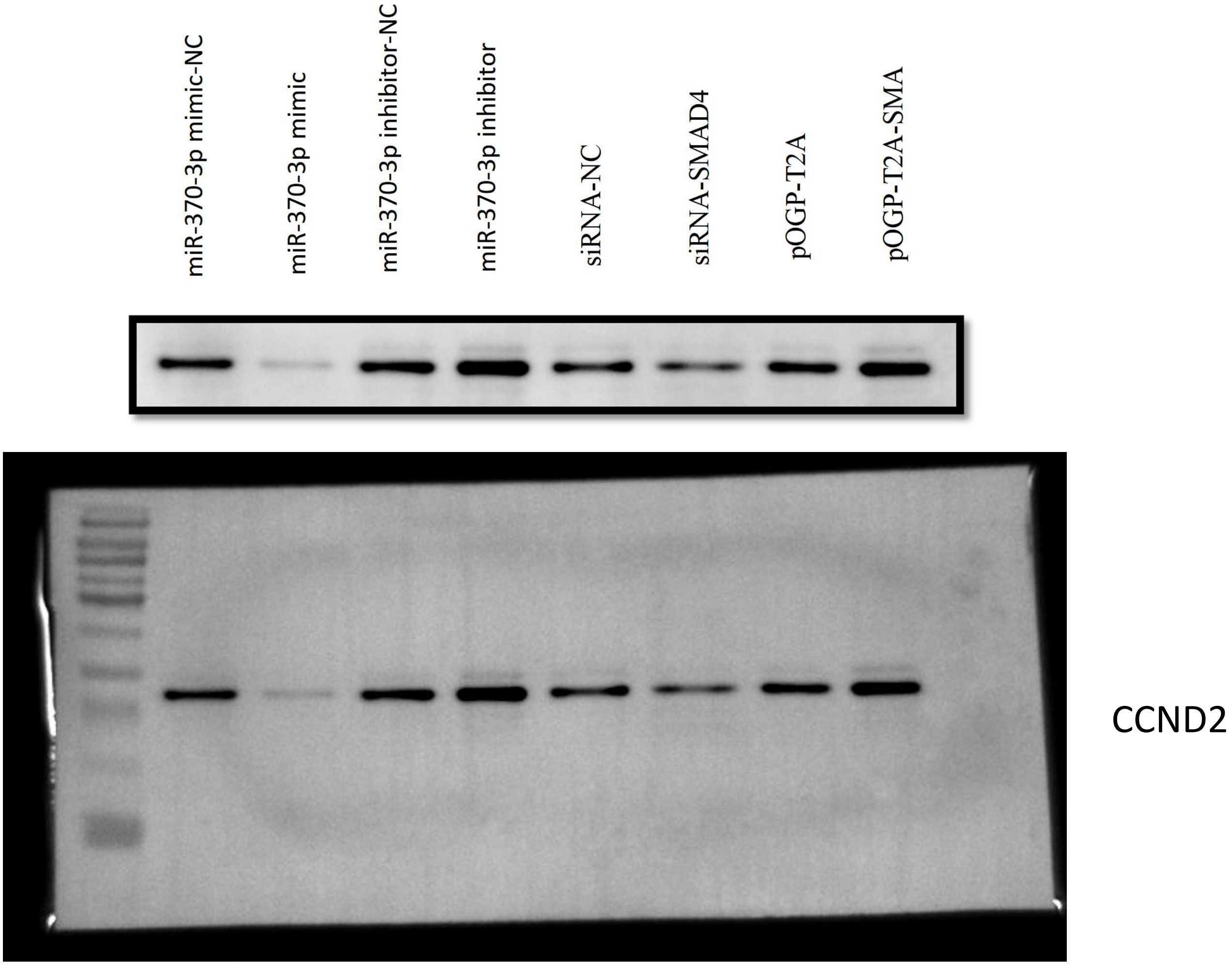
miR-370-3p mimic-NC
miR-370-3p mimic
miR-370-3p inhibitor-NC
miR-370-3p inhibitor
siRNA-NC
siRNA-SMAD4
pOGP-T2A
pOGP-T2A-SMA
CCND2

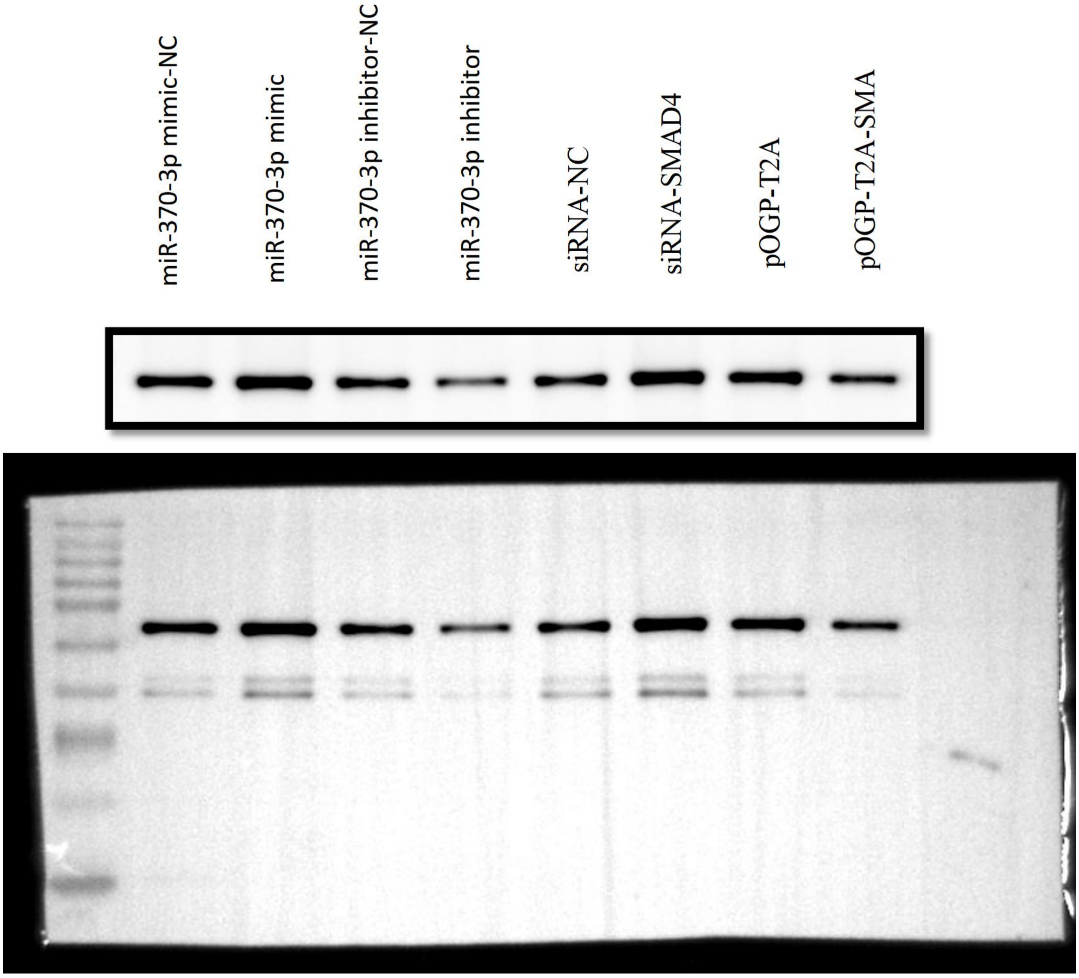
miR-370-3p mimic-NC
miR-370-3p mimic
miR-370-3p inhibitor-NC
miR-370-3p inhibitor
siRNA-NC
siRNA-SMAD4
pOGP-T2A
pOGP-T2A-SMA

C-MYC

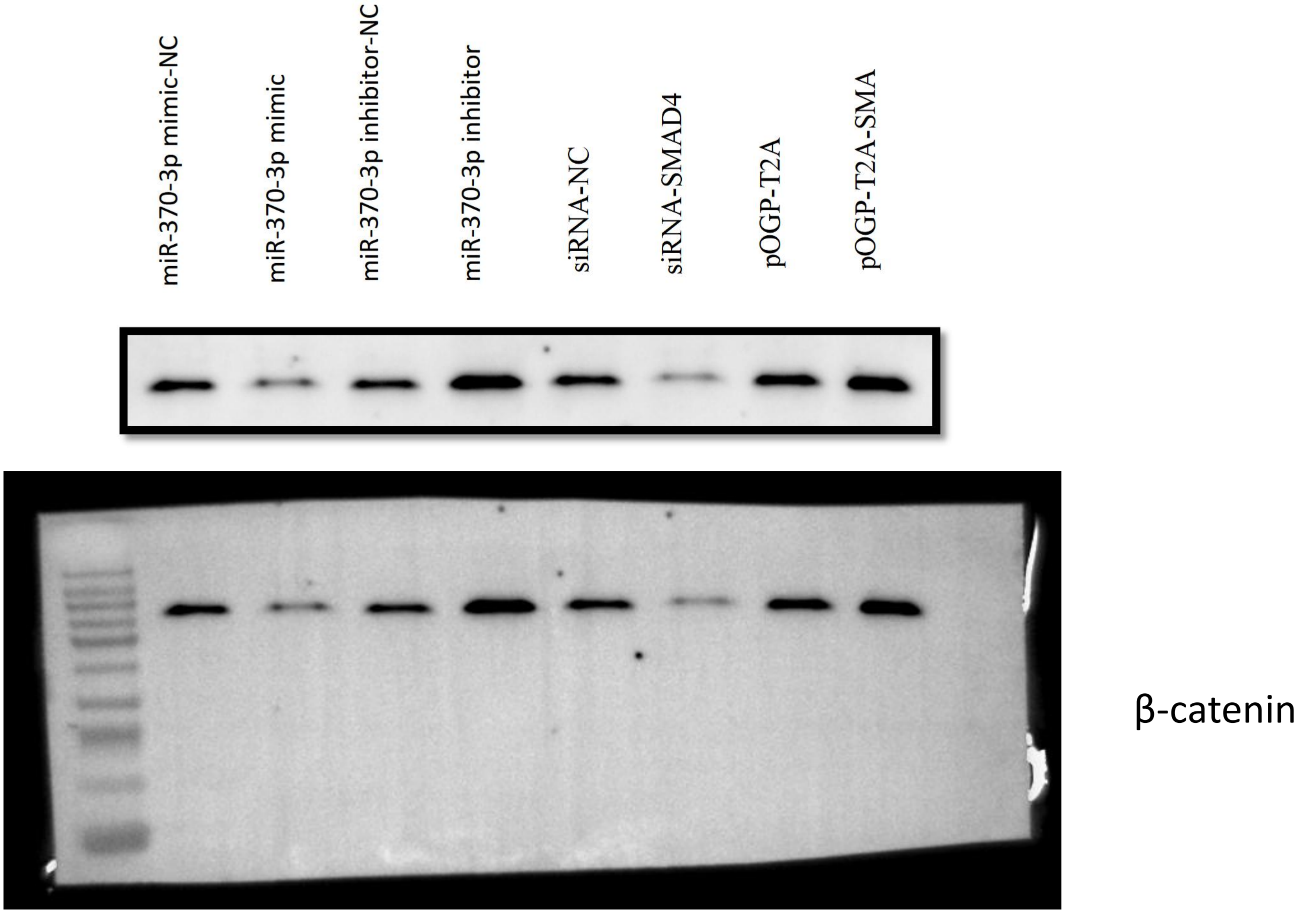
miR-370-3p mimic-NC
miR-370-3p mimic
miR-370-3p inhibitor-NC
miR-370-3p inhibitor
siRNA-NC
siRNA-SMAD4
pOGP-T2A
pOGP-T2A-SMA
β-catenin

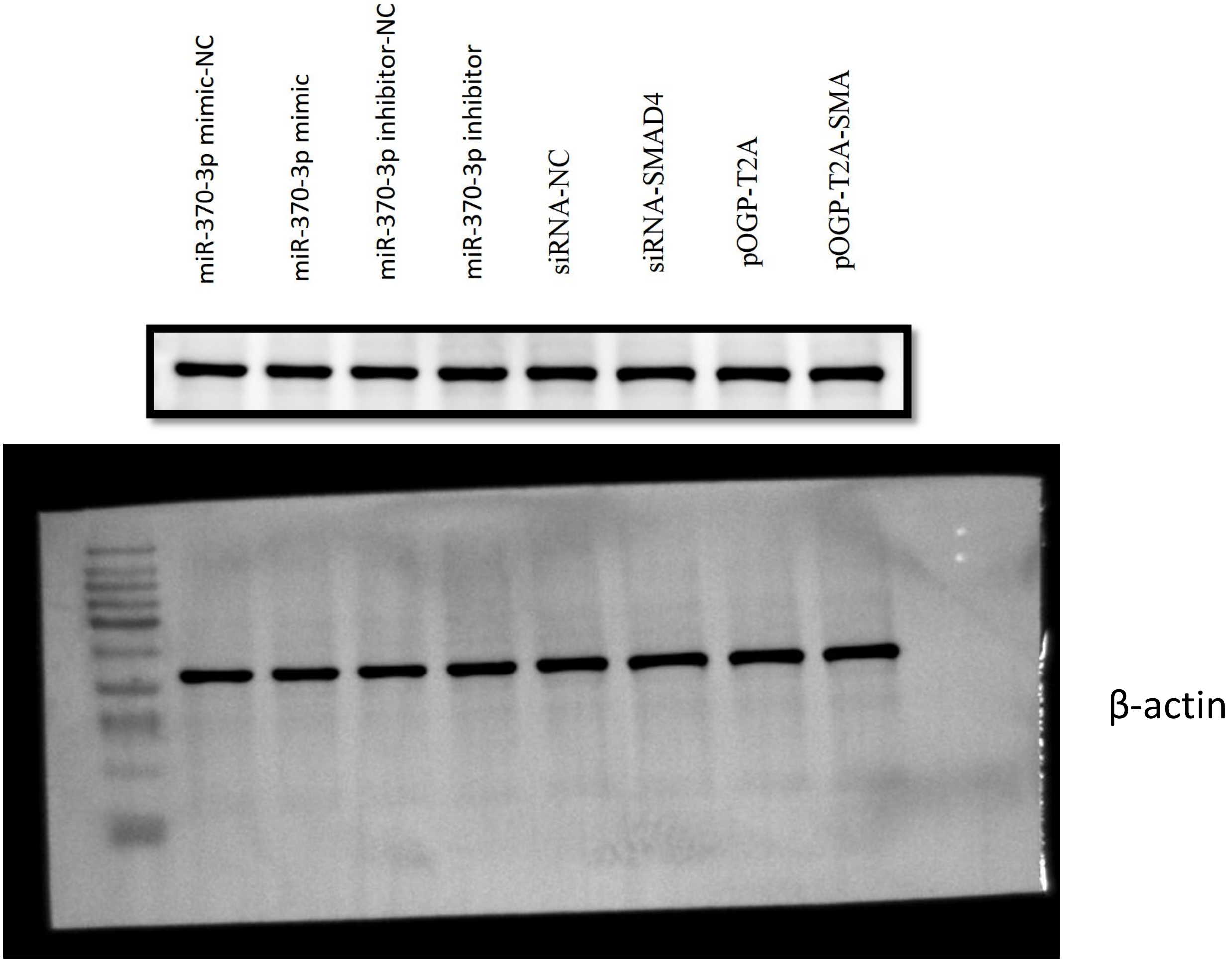

Supplementary Figure S4. Uncropped blots for the experiment shown in Figure 11a describing the expression levels of CCND1,CCND2,C-MYC, β -catenin, β -actin protein.
