# Supplementary figures and images for: miR-370-3p Inhibited the Proliferation of Sheep Dermal Papilla Cells by Inhibiting the Expression of SMAD4
Source: Cells. 2025 May 14;14(10):714. doi: 10.3390/cells14100714 (PMC12110447; doi:10.3390/cells14100714)

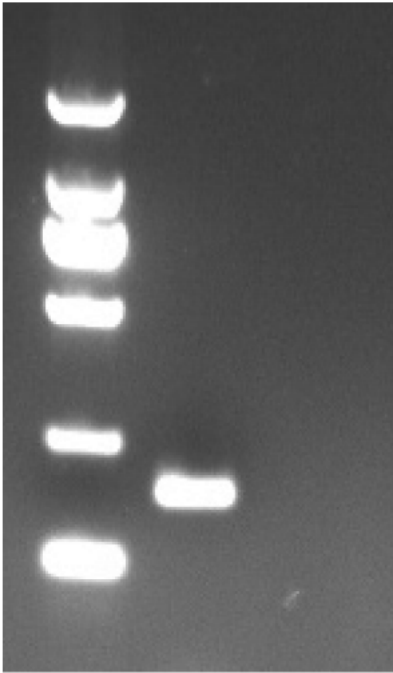

**Supplementary Figure S1** Expression of SMAD4 in DPCs

Supplement: Supplementary file 1 [file cells-14-00714-s001.zip › Supplementary Figure S1.pdf]
